# *miR-3140* suppresses tumor cell growth by targeting *BRD4* via its coding sequence and downregulates the BRD4-NUT fusion oncoprotein

**DOI:** 10.1038/s41598-018-22767-y

**Published:** 2018-03-14

**Authors:** Erina Tonouchi, Yasuyuki Gen, Tomoki Muramatsu, Hidekazu Hiramoto, Kousuke Tanimoto, Jun Inoue, Johji Inazawa

**Affiliations:** 1 Department of Molecular Cytogenetics, Medical Research Institute, Tokyo Medical and Dental University, Tokyo, Japan; 2 Department of Maxillofacial Surgery, Graduate School, Tokyo Medical and Dental University, Tokyo, Japan; 3 Genome Laboratory, Medical Research Institute, TMDU, Tokyo, Japan; 4 Bioresource Research Center, Tokyo Medical and Dental University, Bunkyo-ku, Tokyo, Japan

## Abstract

Bromodomain Containing 4 (BRD4) mediates transcriptional elongation of the oncogene MYC by binding to acetylated histones. BRD4 has been shown to play a critical role in tumorigenesis in several cancers, and the *BRD4-NUT* fusion gene is a driver of NUT midline carcinoma (NMC), a rare but highly lethal cancer. microRNAs (miRNAs) are endogenous small non-coding RNAs that suppress target gene expression by binding to complementary mRNA sequences. Here, we show that *miR-3140*, which was identified as a novel tumor suppressive miRNA by function-based screening of a library containing 1090 miRNA mimics, directly suppressed *BRD4* by binding to its coding sequence (CDS). *miR-3140* concurrently downregulated *BRD3* by bind to its CDS as well as *CDK2* and *EGFR* by binding to their 3’ untranslated regions. *miR-3140* inhibited tumor cell growth *in vitro* in various cancer cell lines, including EGFR tyrosine kinase inhibitor-resistant cells. Interestingly, we found that *miR-3140* downregulated the BRD4-NUT fusion protein and suppressed *in vitro* tumor cell growth in a NMC cell line, Ty-82 cells. Furthermore, administration of *miR-3140* suppressed *in vivo* tumor growth in a xenograft mouse model. Our results suggest that *miR-3140* is a candidate for the development of miRNA-based cancer therapeutics.

## Introduction

The bromodomain and extra-terminal domain (BET) family proteins, including BRD2, BRD3, BRD4, and BRDT, contain two conserved bromodomains that are associated with acetylated lysine in histones, facilitating transcriptional activation as epigenetic readers^1,2^. Among the BET family proteins, BRD4 has been shown to play a critical role in promoting tumor growth in several cancers, including acute myeloid leukemia^3,4^, multiple myeloma^5^, MLL-fusion leukemia^6^, diffuse large B cell lymphoma^7^, triple negative breast cancer^8^, and pancreatic cancer^9^. BRD4 is enriched at super-enhancers of several oncogenes, such as *MYC*, *CCND2*, and *BCL-XL*, in cancer cells, upregulating the transcription of these genes^10^. BRD4 increases the transcription of the other oncogenes *BCL2*, *CDK6*, and *MYC* by binding to the chromatin locus of these genes and recruiting positive transcriptional elongation factor complex (P-TEFb) to the promoter^11^. Thus, BRD4 is thought to be a rational target for cancer therapy^6^.

NUT midline carcinoma (NMC) is a poorly differentiated carcinoma that arises in the midline of the upper aerodigestive tract or the mediastinum^12,13^. NMC is rare, refractory to conventional treatments, and highly lethal, with a median survival period of 6.7–9.5 months^12,13^. The pathogenesis of NMCs involves the *BRD4-NUT* fusion gene, which is caused by a unique chromosome translocation t(15; 19)(q13; p13.1) in the majority of cases, although *BRD3-NUT* fusion by a t(9; 15)(q34; q14), *NSD3-NUT* fusion by a t(8; 15)(p12; q15), and *ZNF532-NUT* fusion by a t(15; 18)(q14; q23) occur in the remaining few cases^13–15^. The translocation breakpoints occur within intron 10 of the *BRD4* gene (19p13.1) and intron 2 of *NUT* (15q14), such that the BRD4-NUT protein contains both acetyl-histone binding bromodomains and the extraterminal domain of BRD4 (i.e., the full functional domain of BRD4)^13^. The BRD4-NUT oncoprotein promotes tumor cell growth through the function of BRD4 as well as that of NUT^16–18^.

The first-generation BET bromodomain inhibitor JQ1 binds to the acetyl-lysine binding pocket of BRD4, and thus, JQ1 depletes not only BRD4 but also BRD4-NUT from chromatin by preventing the binding of BRD4 to chromatin^17,19^. As a result, JQ1 inhibits BRD4-mediated transcription of oncogenes, such as *MYC*^8,20,21^. Several studies have shown that BET bromodomain inhibitors are highly effective against various intractable cancers, including triple negative breast cancer^8^, pancreatic cancer^9^, and NMC^22^, although resistance to BET bromodomain inhibition can be acquired through various mechanisms^8,23^. Several clinical trials using BET bromodomain inhibitors have been started^16,24–26^.

microRNAs (miRNAs) are endogenous small non-coding RNAs of 20–25 nucleotides that decompose or inhibit protein translation of mRNA by binding to complementary mRNA sequences^27,28^. An individual miRNA has multiple target genes, and an individual gene can be targeted by multiple miRNAs^28^. Although many studies have revealed that miRNAs repress gene expression by binding to 3′ untranslated regions (3′UTR) of mRNAs, an increasing body of evidence supports that miRNAs also bind to the coding sequences (CDS) of target mRNAs and that miRNA binding to the CDS of mRNAs can effectively suppress translation^29–31^. Our previous study also revealed that *miR-432–3p* directly targets *KEAP1* via its CDS^32^.

Because a single miRNA can simultaneously repress numerous target genes, miRNA mimics targeting several oncogenes may be useful as therapeutic agents for cancer therapy^33^. In the present study, to investigate the novel candidate miRNAs for the development of miRNA-based cancer therapeutics, we conducted function-based screening using a miRNA library containing 1090 miRNAs. We revealed that *miR-3140*, identified as a novel tumor suppressive miRNA (TS-miRNA), repressed BRD4 directly by binding to its CDS. Furthermore, we showed that *miR-3140* also downregulated the BRD4-NUT oncoprotein in NMC cells. *miR-3140* suppresses other tumor promoting genes, such as *EGFR* and *CDK2*, via 3′UTR. Finally, the effects of *miR-3140* on tumor growth were tested *in vivo*.

## Results

### *miR-3140* was identified as a novel TS-miRNA by function-based miRNA library screening

**Figure 1:**
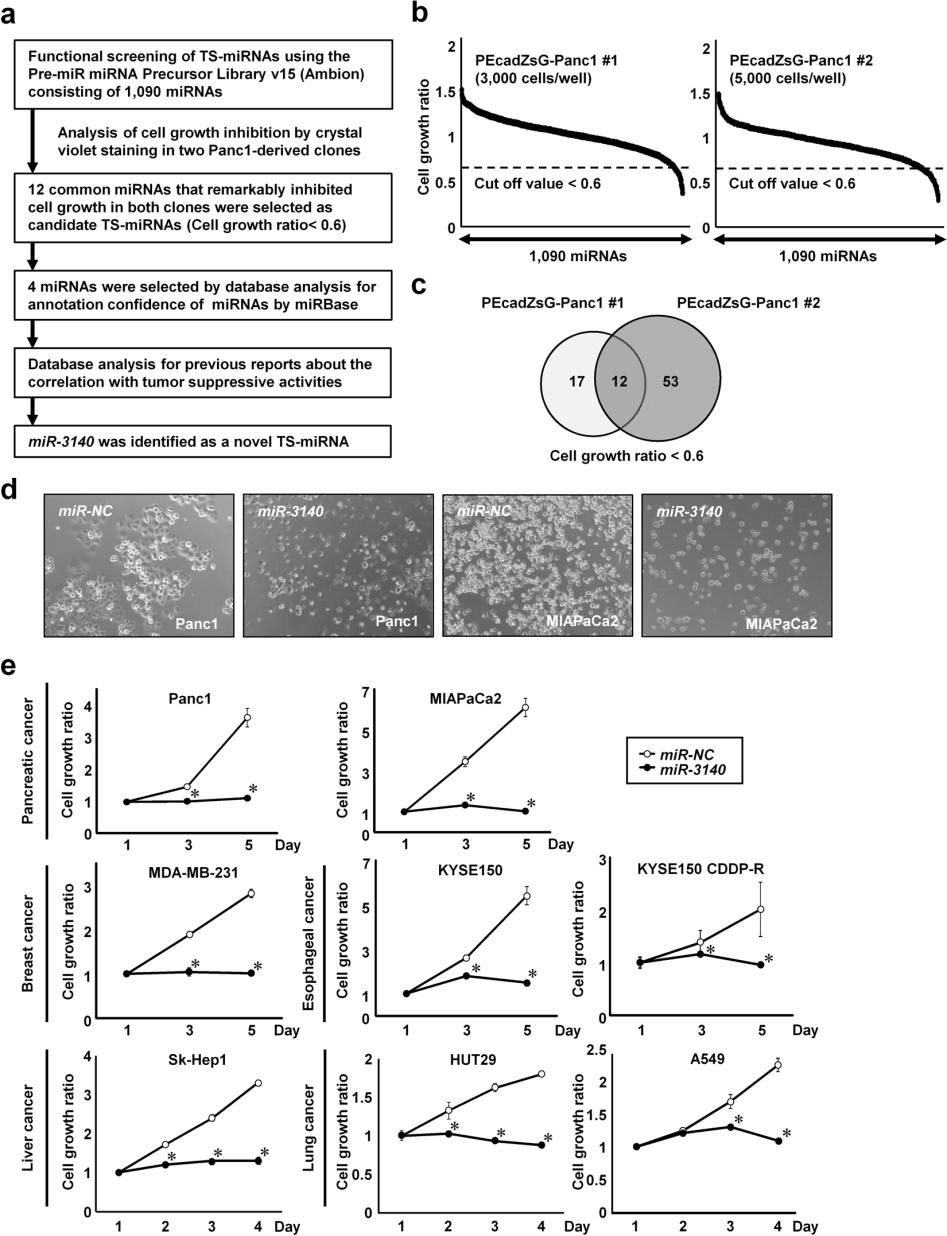
*miR-3140* was identified as a novel tumor suppressive miRNA (TS-miR) by function-based miRNA library screening. (**a**) The strategy used to identify novel TS-miRs in this study. (**b**) Results of function-based miRNA library screening in two Panc1-derived clones (PEcadZsG-Panc1 #1 and PEcadZsG-Panc1 #2 cells), using the Pre-miR miRNA Precursor Library-Human V15 consisting of 1,090 mature human miRNA mimics. The cell growth ratio was assessed with crystal violet staining using a relative ratio normalized based on the cell survival rate of cells transfected with negative control miRNA (*miR-NC*). A value of 0.6 was used to determine the cut-off value for growth inhibition (dotted line). (**c**) The Venn diagram showing the overlap of 12 miRNAs between Panc1 #1 (3000 cells/well) and #2 (5000 cells/well). (**d**) Phase contrast images of Panc1 and MIAPaCa2 cells transfected with 10 nmol/L of *miR-NC* or *miR-3140*. Images were obtained 3 days after transfection. (**e**) Cell growth assay in various types of cancer cells. Pancreatic cell lines (Panc1 and MIAPaCa2), a triple negative breast cancer cell line (MDA-MB-231), esophageal squamous cell carcinoma cell lines (KYSE150 and the cisplatin resistant cell line KYSE150 CDDP-R), a liver cancer cell line (Sk-Hep1), and non-small-cell lung cancer cell lines (HUT29 and A549) were transfected with 10 nmol/L of *miR-NC* or *miR-3140*. The cell growth ratio was assessed with the WST-8 assay based on the relative ratio compared with day 1. Bar, SD for triplicate experiments; *P < 0.05.

To extract novel TS-miRNAs, we performed function-based miRNA library screening from a library containing 1090 miRNA mimics in Panc1 cells. The strategy and brief results of this study are shown in Fig. 1a. In this study, the relative cell growth ratio was determined after transfection of each miRNA in two Panc1-derived clones, PEcadZsG-Panc1 #1 and #2 cells, which were established in our previous study^34^. Figure 1b shows the results of this screening in Panc1 #1 (left) and #2 (right) 72 hours after transfection with each miRNA. We set the criteria for extracting TS-miRNAs (cell growth ratio < 0.6), and then extracted 29 miRNAs from Panc1 #1 and 65 miRNAs from Panc1 #2 cells, respectively (Fig. 1c). Twelve miRNAs that inhibited cell growth in both screening assays were identified as candidate TS-miRNAs (Table 1)^35–38^. Then, 4 miRNAs were extracted according to annotation confidence, which means the certainty of the actual existence of a particular miRNA, determined using miRBase (http://www.mirbase.org/)^39^. Among these 4 miRNAs, little is known about the tumor-suppressive function of *miR-3140*. Thus, we focused on a detailed analysis of *miR-3140*.

**Table 1:** Summary of 12 miRNAs selected as candidates for novel tumor suppressive miRNAs in functional based screening with Pre-miR miRNA Precursor Library v15.

miRNA Precursor	Mature Sequence	Panc1 #1	Panc1 #2	Location	Host gene	Annotation Confidence (miRBase)	Ref^‡^
		Ratio^†^	Ratio^†^				
*hsa-miR-342-5p*	AGGGGUGCUAUCUGUGAUUGA	0.432	0.323	14q32.2	*EVL*	high	^35^
has-miR-449b*	CAGCCACAACUACCCUGCCACU	0.357	0.446	5q11.2	*CDC20*		—
*hsa-miR-608*	AGGGGUGGUGUUGGGACAGCUCCGU	0.487	0.503	10q24.31	*SEMA4G*		^35^
*hsa-miR-634*	AACCAGCACCCCAACUUUGGAC	0.443	0.579	17q24.2	*PRKCA*		^36^
*hsa-miR-671-3p*	UCCGGUUCUCAGGGCUCCACC	0.563	0.467	7q36.1	*CHPF2*	high	^37^
*hsa-miR-876-3p*	UGGUGGUUUACAAAGUAAUUCA	0.407	0.439	9p21.1	*LINGO2*	high	^38^
*hsa-miR-1289*	UGGAGUCCAGGAAUCUGCAUUUU	0.576	0.338	20q11.22	*GDF5*		—
				5q31.1	*FSTL4*		
*hsa-miR-1293*	UGGGUGGUCUGGAGAUUUGUGC	0.580	0.425	12q13.12	*LIMA1*		—
*hsa-miR-3126-3p*	CAUCUGGCAUCCGUCACACAGA	0.582	0.533	2p13.3	*ANTXR1*		—
*hsa-miR-3140*	AGCUUUUGGGAAUUCAGGUAGU	0.508	0.459	4q31.3	*FBXW7*	high	—
*hsa-miR-3165*	AGGUGGAUGCAAUGUGACCUCA	0.533	0.370	11q13.4	*NUMA1*		—
*hsa-miR-3173*		0.551	0.372	14q32.13	*DICER1*		—

^†^Ratio means the cell growth ratio of viable cells as assessed by crystal violet staining 72 hours after transfection with miRNAs, relative to the control transfectants. ^‡^Reference is TS-miRNA.

### *miR-3140* inhibited *in vitro* tumor cell growth in various cancer cell lines

To confirm the growth suppressive effect of *miR-3140* observed in the function-based screening, we evaluated the cell proliferation *in vitro* after transfection with *miR-3140* or *miR-NC* in two pancreatic cancer cell lines, Panc1 and MIAPaCa2 cells, respectively. Consistent with the screening results, overexpression of *miR-3140* significantly inhibited tumor cell growth in both cell lines (Fig. 1d,e). We next investigated whether *miR-3140* suppresses *in vitro* tumor cell growth in various cell lines including triple negative breast cancer (MDA-MB-231), esophageal cancer (KYSE150), liver cancer (Sk-Hep1), and non-small-cell lung carcinoma (HUT29 and A549). We also tested the effects of *miR-3140* in KYSE150 CDDP-R cells, which are resistant to cisplatin^36^. As shown in Fig. 1e, overexpression of *miR-3140* markedly reduced tumor cell growth in all the cancer cell lines we tested. Taken together, *miR-3140* inhibited *in vitro* tumor cell growth.

### *miR-3140* repressed *CDK2* and *EGFR* through 3’UTR interactions

**Figure 2:**
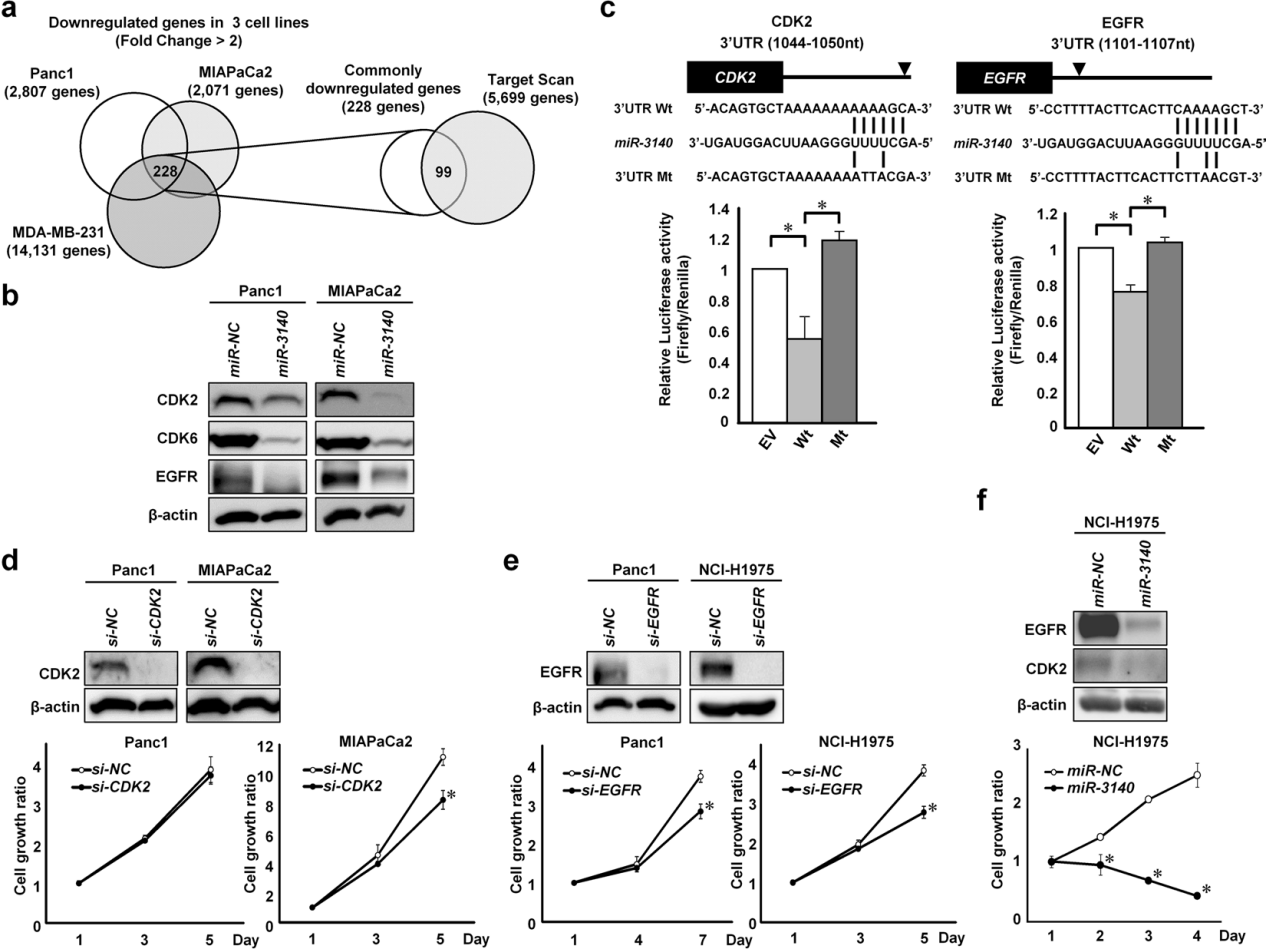
*miR-3140* targeted *CDK2* and *EGFR* by binding their 3′UTR regions. (**a**) Left, identification of downregulated genes after *miR-3140* transfection by a gene expression array. The Venn diagram shows that 228 genes were commonly downregulated (fold change >2) by transfection of *miR-3140* in Panc1, MIAPaCa2, and MDA-MB-231 cells. Right, prediction of candidate target genes regulated by *miR-3140* via their 3′UTR. The Venn diagram shows that 99 genes were predicted as candidate 3′UTR-targets of *miR-3140* by the TargetScan program. (**b**) Western blot analysis of CDK2, CDK6, and EGFR in Panc1 and MIAPaCa2 cells 72 hours after transfection with 10 nmol/L of *miR-NC* or *miR-3140*. (**c**) Luciferase reporter assays. Panc1 cells were transfected with the pmirGLO Dual Luciferase vectors containing wild type (Wt) CDK2 and EGFR or mutant (Mt) 3’UTR target sites of these genes, and after 6 hours, either *miR-NC* or *miR-3140* was additionally transfected. Top, putative binding site of *miR-3140* within the 3′UTR of each gene and mutant sequences. Bottom, results of the luciferase assay; *P < 0.05. (**d**,**e**) Evaluation of the effect of *si-CDK2* or *si-EGFR*. Western blot analysis (top) and cell growth assay (bottom) in indicated cell lines after transfection with 20 nmol/L of negative control siRNA (*si-NC*) or siRNA targeting each gene. (**f**) Evaluation of the effect of *miR-3140* in EGFR-TKI-resistant lung cancer cells. Western blot analysis (top) and cell growth assay (bottom) in the indicated cell line after transfection with 10 nmol/L of *miR-NC* or *miR-3140*. Cell growth ratio was assessed with the WST-8 assay based on the relative ratio compared with day 1. Bar, SD for triplicate experiments; *P < 0.05.

To investigate genes downregulated by *miR-3140*, gene expression array analysis was performed in three cancer cell lines, Panc1, MIAPaCa2, and MDA-MB-231 cells. As shown in a Venn diagram (Fig. 2a, left), the expression levels of 228 genes decreased by more than 2-fold in *miR-3140*-transfected cells compared with *miR-NC*-transfected cells. Among 228 genes, 99 genes were predicted to be direct targets of *miR-3140* according to the TargetScan program (http://www.targetscan.org) because candidate binding-sites for seed sequences of *miR-3140* exist in the 3′UTRs (Fig. 2a, right). Among the overlapping genes, we extracted *CDK2*, *CDK6*, and *EGFR*, which are all known to promote tumor cell growth^40–42^. Western blotting showed that the protein levels of CDK2, CDK6, and EGFR were markedly reduced in *miR-3140*-transfectants of Panc1 and MIAPaCa2 cells, compared with *miR-NC*-transfectants (Fig. 2b). To determine whether *miR-3140* can directly bind to the 3′UTR of *CDK2*, *CDK6* and *EGFR*, we performed luciferase assays using reporter plasmid vectors containing the wild type (Wt) or mutant (Mt) 3′UTR of these genes in Panc1 cells. The luciferase activity of the Wt vectors, except for the 3′UTR of *CDK6* (Supplementary Fig. S1), was decreased compared with the empty vector (EV) in *miR-3140*-transfected cells and completely restored by the Mt vector for *CDK2* and *EGFR* (Fig. 2c). These results indicate that *CDK2* and *EGFR* are direct target genes of *miR-3140* through binding the 3′UTR region, although *CDK6* was downregulated indirectly by *miR-3140*.

To investigate whether CDK2 and EGFR participate in the growth suppressive effects of *miR-3140*, we examined the cell proliferation *in vitro* after transfection with small interfering RNA (siRNA) targeting *CDK2*, *EGFR*, or negative control siRNA. As shown in Fig. 2d, *si-CDK2* significantly reduced tumor cell growth in MIAPaCa2 cells, but not in Panc1 cells. Similarly, *si-EGFR* suppressed cell proliferation in Panc1 cells (Fig. 2e), but not in MIAPaCa2 cells (Supplementary Fig. S2). These results suggested that *CDK2* and *EGFR* contribute to tumor cell growth in a cell-dependent manner. Thus, downregulation of *CDK2* and *EGFR* may partially contribute to *miR-3140*-mediated suppression of tumor cell growth.

### *miR-3140* suppressed tumor cell growth in EGFR tyrosine kinase inhibitor (EGFR-TKI)-resistant lung cancer cells

Since *miR-3140* directly targeted *EGFR*, we next examined whether *miR-3140* overcame the resistance to EGFR-TKIs. *EGFR* is one of the most frequently mutated “driver” genes in non-small cell lung carcinoma (NSCLC) and EGFR-TKIs are used for the treatment of *EGFR*-mutated-NSCLC. Therefore, we examined the effects of *miR-3140* in *EGFR*-mutated-NSCLC cells. In agreement with past reports, HUT29 cells, which harbor the EGFR L858R mutation, were sensitive to the EGFR-TKIs gefitinib and erlotinib, whereas NCI-H1975 cells, which harbor the EGFR L858R/T790M double mutation, were resistant to gefitinib and erlotinib (Supplementary Fig. S3a,b)^43^. Both *miR-3140* and *si-EGFR* reduced the expression of EGFR in NCI-H1975 cells, resulting in the suppression of *in vitro* tumor cell growth in NCI-H1975 cells (Fig. 2e,f). These results suggested that *miR-3140* may overcome the acquired resistance to EGFR-TKIs at least in part by suppressing mutant EGFR in NSCLC cells.

### Suppression of BET family genes inhibited tumor cell growth

**Figure 3:**
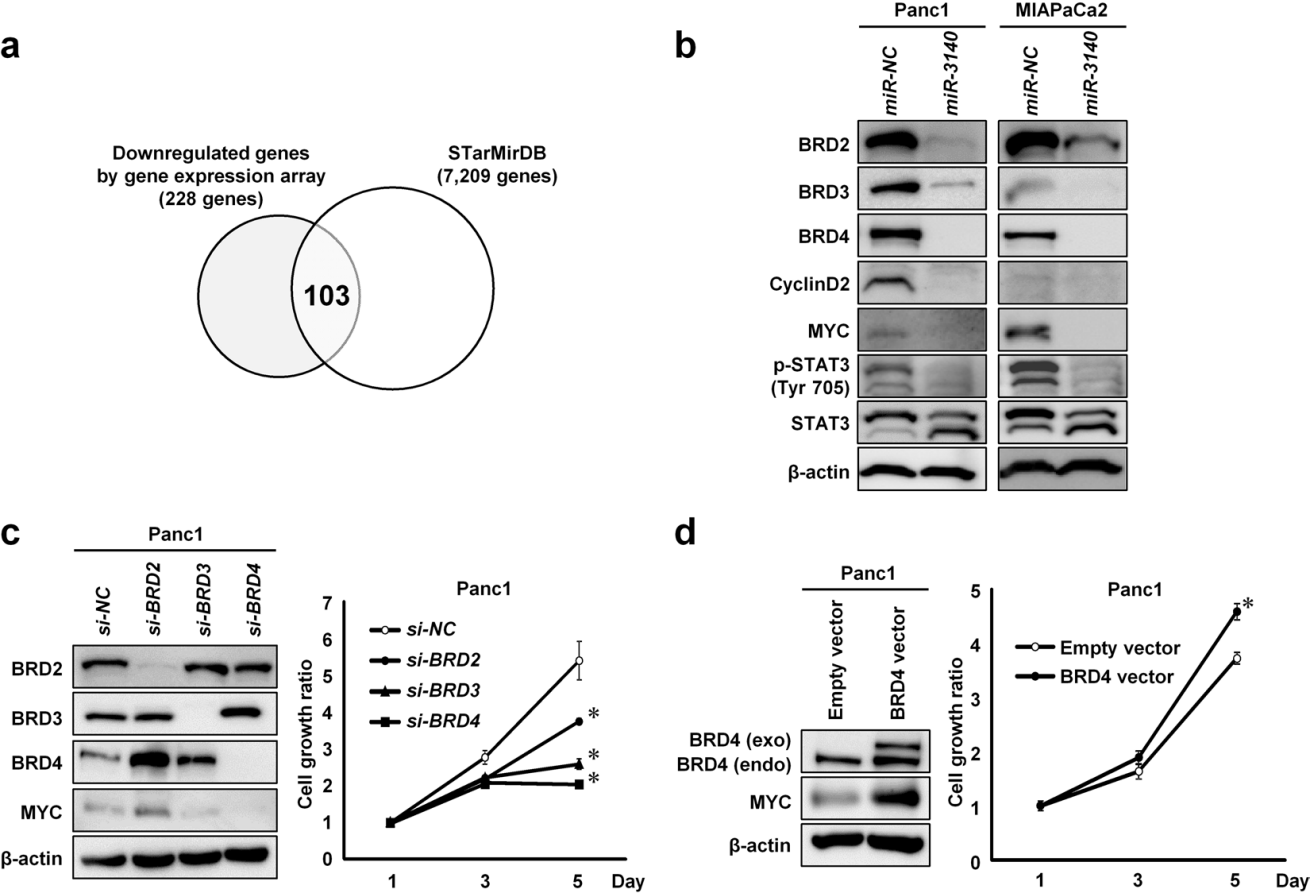
*miR-3140* downregulated BET family genes. (**a**) Prediction of candidate targets regulated by *miR-3140* via their coding sequences (CDS). The Venn diagram shows that 103 genes were predicted as candidate CDS-targets of *miR-3140* by the STarMirDB program. (**b**) Western blot analysis of BET family proteins (BRD2, BRD3, and BRD4) and downstream targets of BRD4 (Cyclin D2, MYC, and p-STAT3) in Panc1 and MIAPaCa2 cells 72 hours after transfection with 10 nmol/L of *miR-NC* or *miR-3140*. (**c**,**d**) Effects of knockdown of BET family proteins (**c**), or overexpression of BRD4 (**d**) on cell growth in Panc1 cells. Cells were transfected with 20 nmol/L of *si-NC* or siRNA targeting BET family proteins (**c**), and empty vector or BRD4-HaloTag expression vector (**d**). Left, western blot analysis of indicated proteins in Panc1 cells 72 hours after transfection. Right, results of the cell growth assay. The cell growth ratio was assessed with the WST-8 assay based on the relative ratio compared with day 1. Bar, SD for triplicate experiments; *P < 0.05.

Because the effects of *miR-3140* on *in vitro* tumor cell growth were much greater than those of *si-CDK2* and *si-EGFR* (Figs 1e and 2d–f), concurrent downregulation of additional target genes may be necessary for the induction of *miR-3140*-mediated inhibition of tumor cell growth. A growing body of evidence suggested that coding sequence (CDS) of mRNA could be directly targeted by a miRNA-containing RISC complex^29–31^. Thus, we explored additional target genes of *miR-3140* using the STarMirDB database (http://sfold.wadsworth.org)^44^, which predicts miRNA target genes based on CDS binding. Among the 228 genes downregulated by *miR-3140* in 3 cell lines (Fig. 2a), candidate binding-sites for the seed sequence of *miR-3140* exist in the CDS of 103 genes (Fig. 3a, Supplementary Table S1). From these 103 overlapping genes, we extracted *BRD4* as a candidate target gene of *miR-3140*, because BRD4 has been shown to play a critical role in promoting tumor growth in several cancers through upregulates the transcription of oncogenes, including *MYC* and *CCND2*. Our gene expression array analysis showed that *miR-3140* downregulated the expression of *MYC* and *CCND2* as well as *BRD4* in Panc1 cells, suggesting that BRD4 plays a critical role for tumor cell growth in Panc1 cells. As shown in Fig. 3b, *miR-3140* markedly reduced BRD4 protein expression. Furthermore, we found that other BET family genes, BRD2 and BRD3, together with genes for the downstream signaling pathways of BRD4, MYC, phosphorylated STAT3 and Cyclin D2^10,20^, were significantly reduced by *miR-3140* (Fig. 3b). Transfection of siRNA targeting each BET family gene, especially *si-BRD4*, markedly inhibited tumor cell growth (Fig. 3c, Supplementary Fig. S4). Conversely, overexpression of *BRD4* promoted cell proliferation compared to transfection with an empty vector (Fig. 3d). Taken together, downregulation of BRD4 contributed to *miR-3140*-mediated suppression of tumor cell growth.

### *miR-3140* repressed *BRD4* and *BRD3* by directly binding to the CDS region

**Figure 4:**
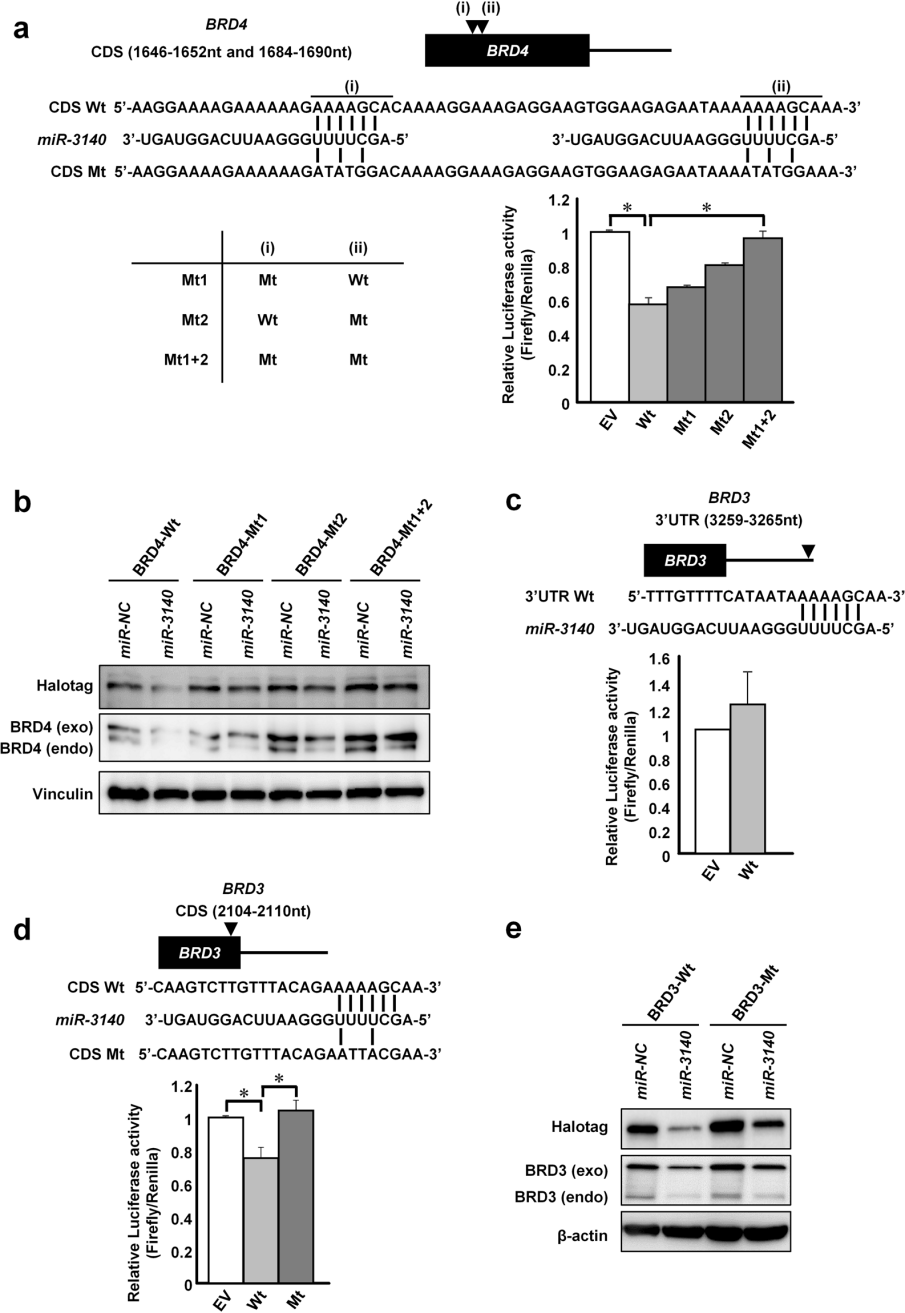
*miR-3140* directly targeted *BRD4* and *BRD3* by binding their coding regions. (**a**) Luciferase reporter assay. MIAPaCa2 cells were transfected with the pmirGLO Dual Luciferase vectors containing Wt or Mt *BRD4*, or empty vector (EV), and after 6 hours, either *miR-NC* or *miR-3140* was additionally transfected. Top, putative binding sequence of *miR-3140* within the CDS of *BRD4* and mutant sequences are indicated. Bottom, the results of luciferase assay; *P < 0.05. (**b**) Western blot analysis of BRD4 in MIAPaCa2 cells. Cells were co-transfected with the Wt or Mt BRD4 expression vector, and after 24 hours, either 10 nmol/L of *miR-NC* or *miR-3140* was additionally transfected. (**c**,**d**) Luciferase reporter assays. Panc1 cells were transfected with a reporter plasmid (Wt of *BRD3* 3′UTR or EV (**c**), or Wt, Mt of *BRD3* CDS or EV (**d**)) and after 6 hours, either *miR-NC* or *miR-3140* was additionally transfected. Top, putative binding sequences of *miR-3140* within the *BRD3* 3′UTR (**c**) and the *BRD3* CDS (**d**). Bottom, results of the luciferase assay; *P < 0.05. (**e**) Western blot analysis of BRD3 in Panc1 cells. Cells were co-transfected with the Wt or Mt BRD3 expression vector, and after 24 hours, either 10 nmol/L of *miR-NC* or *miR-3140* was additionally transfected.

To examine whether *miR-3140* can directly bind to the CDS of *BRD4*, we first performed luciferase assays using reporter plasmid vectors containing Wt or Mt seed sequences in the CDS. Because two candidate binding-sites for the seed sequence of *miR-3140* exist within the CDS of *BRD4*, three mutant constructs (Mt1, Mt2 and Mt1 + 2) were established (Fig. 4a). The luciferase activity of the Wt vector was decreased compared with EV in *miR-3140* transfected cells and partially recovered with each Mt1 or Mt2 vector and completely restored with the Mt1 + 2 vector (Fig. 4a). We next examined whether *miR-3140* suppressed the expression of BRD4 by binding to these positions within the CDS. Synonymous mutations were generated at each site of the Halotag-BRD4 protein (Supplementary Fig. S5a). Each of those Wt and Mt vectors were transfected into MIAPaCa2 cells followed by transfection of *miR-NC* or *miR-3140*, respectively. Whereas the expression level of exogenously expressed BRD4-Wt was reduced in the *miR-3140*-transfected cells, the *miR-3140*-induced reduction of exogenous BRD4 was restored by all three patterns of each mutation at binding-sites for the seed sequence of *miR-3140* (Fig. 4b). These combined data suggested that *miR-3140* can downregulate BRD4 expression by directly targeting its CDS.

Similarly, we examined whether *BRD3* was also a candidate target gene of *miR-3140*. Candidate binding-sites for the seed sequence of *miR-3140* exist within both the CDS and the 3′UTR of the *BRD3* gene. The luciferase activity of the 3′UTR-Wt vector did not decrease, but that of the CDS-Wt vector was decreased compared with EV in *miR-3140* transfectant, and this decrease was completely recovered by the CDS-Mt vector (Fig. 4c,d). Synonymous mutations were generated at the CDS site of the Halotag-BRD3 protein (Supplementary Fig. S5b). While the expression level of exogenously expressed BRD3-Wt was decreased in the *miR-3140*-transfected cells, this *miR-3140*-induced decrease in exogenous BRD3 was restored by the mutations at a binding-site for the seed sequence of *miR-3140* (Fig. 4e), suggesting that *miR-3140* can downregulate BRD3 expression by targeting its CDS as well as BRD4. Taken together, *miR-3140* targets *BRD4* and *BRD3* through their binding CDS regions.

### *miR-3140* downregulated the BRD4-NUT fusion protein

**Figure 5:**
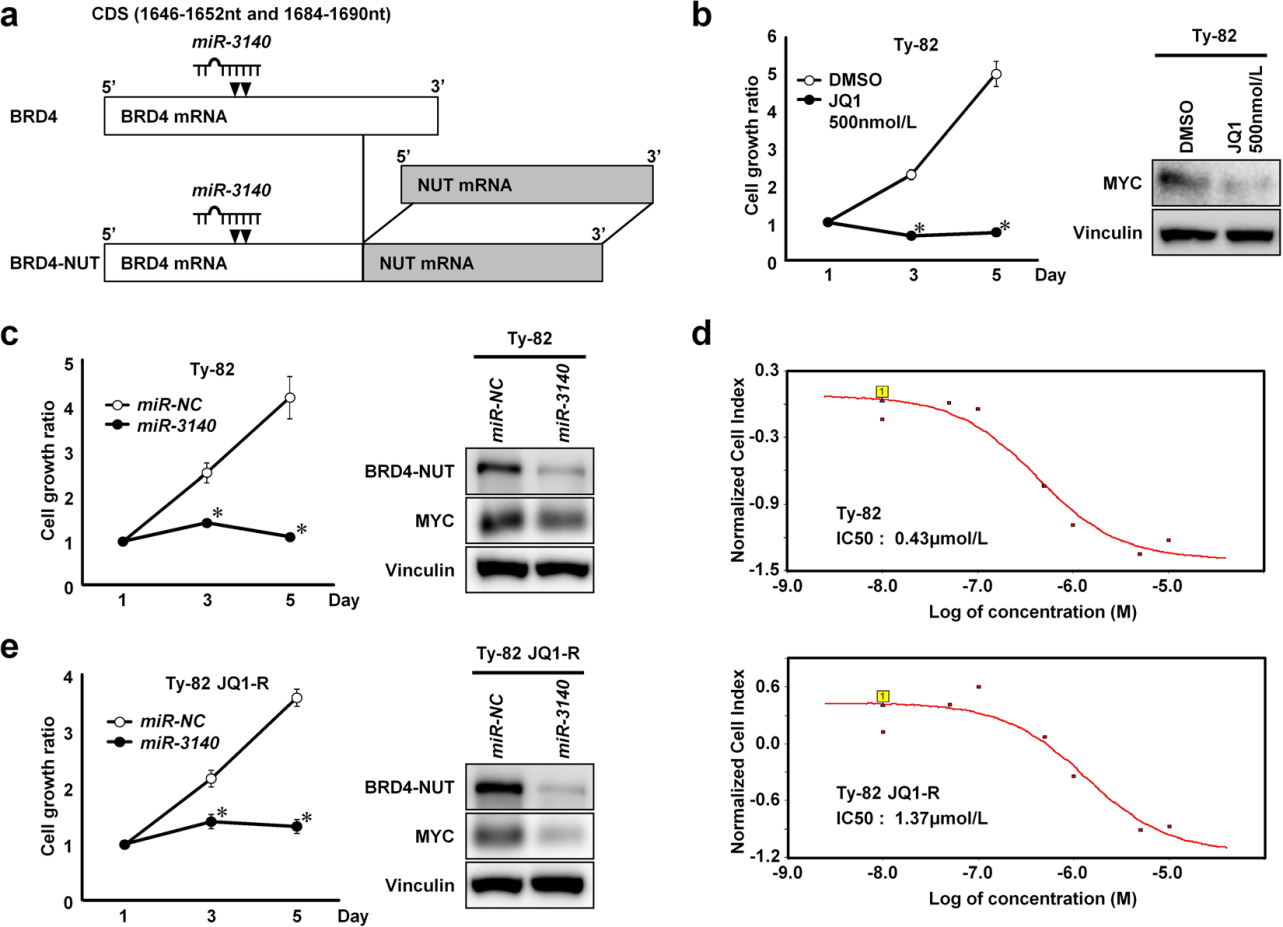
*miR-3140* downregulates the BRD4-NUT fusion protein. (**a**) Schema of the relationship between mRNA of the *BRD4-NUT* fusion gene in NUT midline carcinoma and the location of *miR-3140* target sites. (**b**) Effects of the BET bromodomain inhibitor JQ1 in Ty-82 cells. Left, the results of the cell growth assay. The cell growth ratio was assessed with the WST-8 assay based on the relative ratio compared with day 1. Bar, SD for triplicate experiments; *P < 0.05. Right, western blot analysis of MYC in Ty-82 cells 3 hours after transfection. (**c**,**e**) Left, cell growth assay in a NUT midline carcinoma cell line, Ty-82 (**c**) and its JQ1-resistant cells (Ty-82 JQ1-R) cells (**e**) which were transfected with 10 nmol/L of *miR-NC* or *miR-3140*. Cell growth ratio was assessed with the WST-8 assay based on the relative ratio compared with day 1. Bar, SD for triplicate experiments; *P < 0.05. Right, western blot analysis of the BRD4-NUT fusion protein and MYC in Ty-82 (**c**) and Ty-82 JQ1-R cells (**e**) 72 hours after transfection with 10 nmol/L of *miR-NC* or *miR-3140*. (**d**) Dose response curve of JQ1 for Ty-82 (upper) and Ty-82 JQ1-R cells (lower) at 50 hours following treatment with JQ1. Cell indexes were normalized with the last time point before JQ1 treatment.

Based on the results that *miR-3140* directly targets the CDS of *BRD4*, we investigated whether *miR-3140* suppresses the *BRD4-NUT* fusion gene. The *BRD4-NUT* fusion is caused by t(15:19) translocation and drives NUT midline carcinoma (NMC), a rare, highly lethal cancer (Fig. 5a)^12^. Consistent with past reports^21,45^, JQ1 reduced MYC and significantly suppressed *in vitro* tumor growth of Ty-82 cells, a NMC cell line, which harbor t(15;19) bearing the BRD4-NUT fusion gene (Fig. 5b)^46^. As shown in Fig. 5c, *miR-3140* repressed the expression of the BRD4-NUT fusion protein and its downstream target MYC in Ty-82 cells. As a result, *miR-3140* effectively suppressed *in vitro* tumor cell growth of Ty-82 cells (Fig. 5c).

Next, to test whether *miR-3140* can suppress tumor cell growth of JQ1-resistant cells, JQ1-resistant cells (Ty-82 JQ1-R) were generated from Ty-82 cells (IC50: 0.43 µM in Ty-82 cells, 1.37 µM in Ty-82 JQ1-R cells; Fig. 5d). To determine whether Ty-82 JQ1-R cells have developed resistance by acquiring BRD4-NUT-dependence or not, we examined the effects of JQ1 in Ty-82 JQ1-R cells. The expression of MYC, a downstream effector of BRD4-NUT, was not suppressed sufficiently by JQ1 treatment in Ty-82 JQ1-R cells, whereas the expression of MYC was suppressed by JQ1 treatment in a dose-dependent manner in Ty-82 cells (Supplementary Fig. S6a). Knockdown of BRD4-NUT using RNA interference downregulated the expression of MYC and suppressed cell growth both in Ty-82 JQ1-R cells (Supplementary Fig. S6b). These results suggested that JQ1 could not block the effects of BRD4-NUT sufficiently in Ty-82 JQ1-R cells, although the mechanism is unknown. *miR-3140* downregulated the expression of BRD4-NUT fusion protein and MYC, and suppressed tumor cell growth in Ty-82 JQ1-R cells as well as Ty-82 cells (Fig. 5c,e). These combined data suggested that the resistance of JQ1 is, at least in part, dependence of BRD4-NUT/MYC pathway. Thus, our results suggested that *miR-3140* inhibited cell growth in Ty-82 JQ1-R cells by targeting BRD4-NUT.

### *miR-3140* suppressed tumor growth in a xenograft mouse model

**Figure 6:**
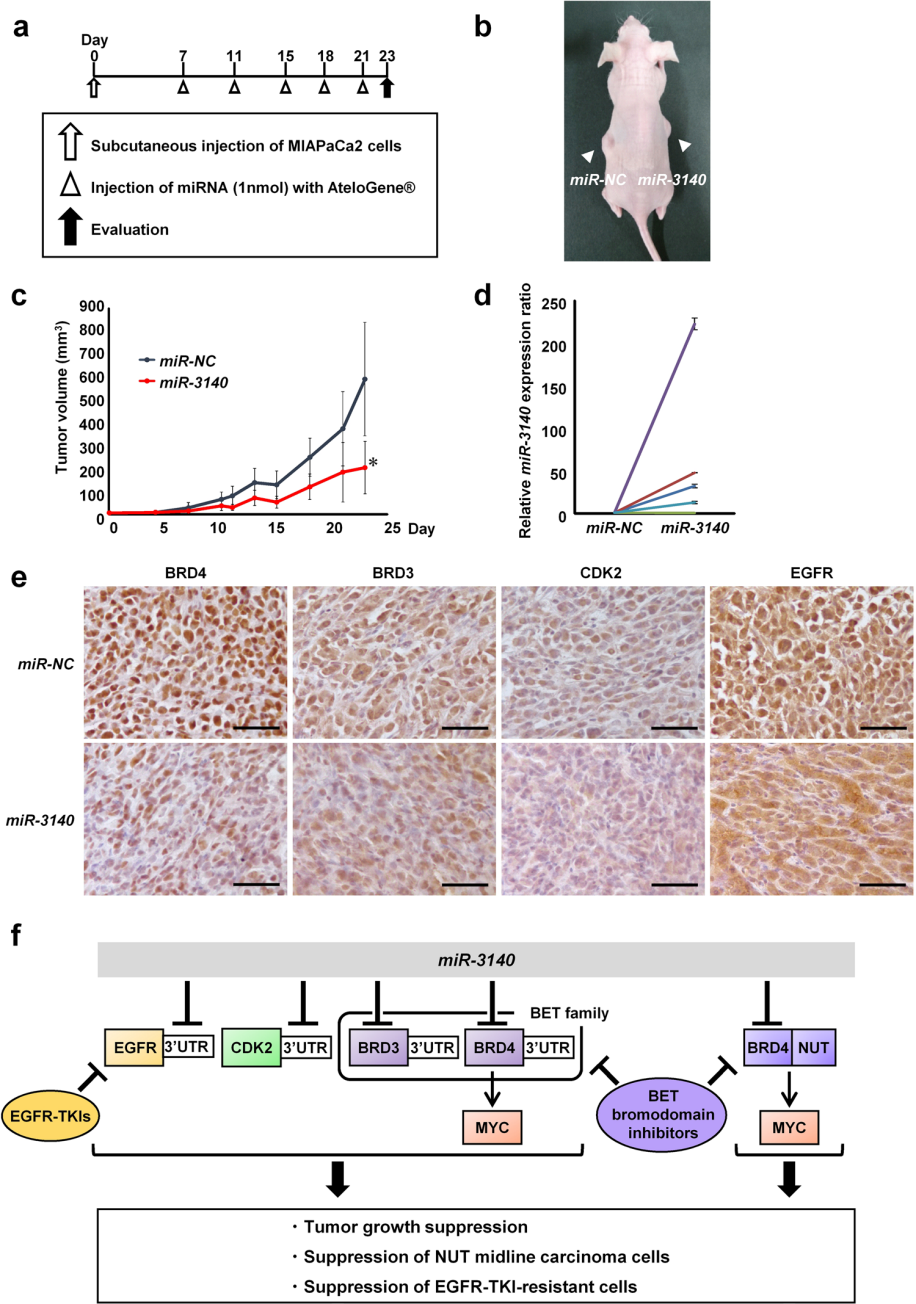
Therapeutic effects of *miR-3140* for tumor growth *in vivo*. (**a**) The experimental schedule for *miR-3140* treatment in nude mice which were subcutaneously inoculated with MIAPaCa2 cells. (**b**) The representative image of tumor-bearing nude mice at 23 days after the inoculation of MIAPaCa2 cells. Tumors are denoted by arrowheads. (**c**) Tumor growth curves of xenograft mouse models treated with *miR-NC* or *miR-3140* (n = 5, each). Tumor volume was calculated using the following formula: (shortest diameter) ^2^ × (longest diameter) × 0.5. Bar, SD for 5 mice; *P < 0.05. (**d**) Expression analysis of *miR-3140* in resected tumors. The expression level of *miR-3140* was measured by qRT-PCR using the relative ratio normalized based on the expression of *RNU6B*. Each experiment was performed in duplicate. Bar, SD. (**e**) Representative images of immunohistochemical staining for BRD4, BRD3, CDK2 and EGFR in resected tumors. Scale bar, 50 μm $.$ (**f**) Schematic models for the mechanism by which *miR-3140* suppresses tumor growth.

We next examined the therapeutic effect of *miR-3140* through the local administration of dsRNA mimicking miRNA around MIAPaCa2-derived subcutaneous tumors in nude mice. *miR-NC* (left) or *miR-3140* (right) were administered into the subcutaneous space around tumors 5 times (7, 11, 15, 18, 21 days after the injection of MIAPaCa2 cells; Fig. 6a). Administration of miRNA did not produce any adverse consequences, such as body weight loss or local damage. As a result, tumors treated with *miR-3140* at 23 days after the injection of MIAPaCa2 cells were significantly smaller than tumors treated with *miR-NC* (Fig. 6b,c, Supplementary Fig. S7a,b). We confirmed that the expression of *miR-3140* was significantly high in the *miR-3140*-treated tumors compared to *miR-NC*-treated tumors by qRT-PCR (Fig. 6d). Furthermore, immunohistochemical staining showed that the expression of BRD4, BRD3, CDK2, and EGFR, the targets of *miR-3140*, were reduced in the resected tumors treated with *miR-3140* (Fig. 6e, Supplementary Fig. S8). Although other targets of *miR-3140* may also participate in the tumor suppressive activity, *miR-3140* inhibited tumor growth *in vivo* at least in part by suppressing *BRD4*, *BRD3*, *CDK2* and *EGFR*.

## Discussion

Here, we identified *miR-3140* as a novel TS-miRNA by function-based miRNA library screening. As summarized in Fig. 6f, we showed that *miR-3140* inhibited tumor cell growth in various cancer cells both *in vitro* and *in vivo* at least in part by directly targeting *BRD4*, *BRD3*, *CDK2*, and *EGFR*. Furthermore, we revealed that *miR-3140* suppressed the BRD4-NUT oncoprotein in NMC cells and that *miR-3140* inhibited *in vitro* tumor cell growth in NMC cells.

We revealed that *miR-3140* directly suppressed *EGFR* and *CDK2* via 3′UTR interaction. Both EGFR and CDK2 play a role in cancer progression. EGFR is activated by gain-of-function mutations or amplification in several cancers including lung, head and neck, ovary, colon, and esophagus^43^. Small molecule inhibitors, such as gefitinib and erlotinib, are used in lung cancer patients who have mutations on *EGFR*^43^. We showed that *miR-3140* suppressed *in vitro* tumor cell growth by directly reducing EGFR expression in NCI-H1975 cells, which are EGFR-TKI resistant due to EGFR L858R/T790M double mutations^43,47,48^. Although *in vivo* experiments are needed to confirm the effects of *miR-3140* on EGFR-TKI-resistant cells, our results suggest that *miR-3140* may overcome the acquired resistance to EGFR-TKIs in lung cancer. *CDK2* is another target of *miR-3140*. The catalytic activity of CDK2-CyclinE complexes is hyperactivated in several cancers by *Cyclin E* amplification, or a loss-of-function mutation of *FBXW7*, a ubiquitin ligase for Cyclin E degradation, although *CDK2* mutations are rare in human cancers^49^. Thus, suppression of CDK2 by *miR-3140* may contribute to the inhibition of tumor growth in some cancers.

Second, we identified that *miR-3140* suppressed *BRD4* and *BRD3* through binding to their CDS. Because BET bromodomain inhibitors suppressed BET family-mediated transcription of the oncogene *MYC*, they were reported to be promising agents for many cancers^5,22^. Several BET inhibitors have entered into clinical trials in some cancers, including NMC^16,24,25^. However, development of resistance to BET inhibition has been reported, in the same way as occurs in the other molecular targeted drugs. For example, BET inhibitor resistance is associated with increased BRD4 phosphorylation mediated by casein kinase 2 (CK2) in triple negative breast cancer cells^8^. We showed that *miR-3140* suppressed the expression of BRD4-NUT in NMC cells. In the *BRD4-NUT* fusion gene, the translocation breakpoints of *BRD4* is within intron 10^50^, and the binding sequence of *miR-3140* exists within exon 9 of *BRD4*. Thus, *miR-3140* can directly target the *BRD4-NUT* fusion gene and consequently, *miR-3140* suppressed *in vitro* tumor cell growth of Ty-82 cells as well as JQ1. Unfortunately, Ty-82 cells hardly formed subcutaneous tumors in our xenograft model. Further *in vivo* studies of the effect of *miR-3140* for NMC cells are needed for future work.

Our data suggested that *miR-3140* could suppress *in vitro* tumor cell growth of JQ1-resistant Ty-82 cells. Direct suppression of BRD4-NUT and concurrent downregulation of other tumor promoting genes such as *EGFR* and *CDK2* by *miR-3140* may potentially overcome resistance to BET inhibitors in NMC cells, although further studies are needed to clarify the acquired resistance of BET inhibitiors. On the other hand, *miR-3140* might not suppress BRD3-NUT, since the translocation breakpoint of *BRD3* in *BRD3-NUT* fusion gene is within intron 9^51^, and the binding sequence of *miR-3140* exists within exon 10 of *BRD3*.

It was reported that the expression of BRD4 were increased by gemcitabine treatment in Panc1 and MIAPaCa2 cells, and the combination of BRD4 silencing and gemcitabine treatment had a synergistic effect on the chemotherapeutic efficacy^52^. A combined treatment of gemcitabine with *miR-3140* also may increase gemcitabine sensitivity in pancreatic cancer.

In primary samples, we did not observe a correlation between the expression levels of *miR-3140* and overall survival in pancreatic cancer, breast cancer, or acute myeloid leukemia according to the TCGA database (Supplementary Fig. S9a–c). This is because the *miR-3140* expression levels are very low in the majority of primary tumor samples, although *miR-3140* is predicted to exist in human tissue according to the miRBase database. Further investigation of the expression pattern of *miR-3140* in non-tumor tissues is required.

Because of recent advances in clinical tumor sequencing and developments in small molecule inhibitors, molecular targeted therapy is one of the key therapeutic strategies along with conventional chemotherapy, radiation therapy, and immunotherapy. However, acquired resistance to molecular targeted drugs is a major problem for cancer treatment^48^. Reduction of the molecular target itself and other tumor promoting targets by a miRNA-based therapy may contribute to overcome the drug tolerance that develops against molecularly targeted drugs. In this study, our findings suggest that *miR-3140* suppresses tumor cell growth not only in various cancer cells, but also in EGFR-TKIs-resistant cells and JQ1-resistant cells. Although further *in vivo* studies of drug delivery systems and possible off-target effects are needed, *miR-3140* may be a candidate for the development of miRNA-based cancer therapeutics.

## Materials and Methods

### Cell culture

Panc1 and MIAPaCa2 cells were cultured in Dulbecco’s Modified Eagle Medium containing 10% Fetal Bovine Serum (FBS). MDA-MB-231 cells from the American Type Culture Collection (Manassas, VA) were maintained in L-15 medium containing 10% FBS. KYSE150 cells, a gift from Dr. Shimada Y (Toyama University)^53^, NCI-H1650, NCI-H1975, A549, HUT29 from ATCC, the NUT midline carcinoma cell line Ty-82 from JCRB, and 11–18 were maintained in RPMI1640 medium containing 10% FBS. The KYSE150 CDDP-resistant cell line (KYSE150 CDDP-R), which is resistant to cisplatin, was generated previously^36^. Sk-Hep1 cells were maintained in Eagle’s Minimum Essential Medium containing 10% FBS. All cell lines were maintained at 37 °C with 5% CO2. The all experiments were carried out in accordance with the approved guidelines and regulations (2010-5-4, 2016-011C2).

### Function-based miRNA screening

Two Panc1-derived clones (PEcadZsG-Panc1 #1 and #2 cells)^34^ were seeded on 96-well plates. After 24 hours, each clone was transfected in duplicate with each of the 1090 dsRNAs from the Pre-miR miRNA Precursor Library-Human V15 (Thermo Fisher Scientific, CA) or a negative control miRNA (*miR-NC*) using an RNA concentration of 10 nmol/L. After 72 hours, viable cell number was assessed by the crystal violet staining assay. The results were normalized to the cell numbers of cells transfected with *miR-NC*.

### Transfection of miRNAs and siRNAs

The dsRNA mimicking mature human *miR-3140-3p* (MC17496) and nonspecific control miRNA (negative control #1) were purchased from Thermo Fisher Scientific. The SMARTpool siRNA for *BRD2* (M-004935-02), *BRD3* (M-004936-01), *BRD4* (M-004937-02), *CDK2* (M-003236-04), *EGFR* (L-003114-00), nonspecific control siRNAs, and a set of 4 siRNA for *BRD4* (MQ-004937-02) were from GE Healthcare (Buckinghamshire, UK). Each SMARTpool siRNA consists of 4 siRNA duplexes designed to target different regions of the same gene. mRNA sequence. miRNAs and siRNAs were transfected individually into cells at the indicated concentrations using Lipofectamine RNAiMAX (Thermo Fisher Scientific) according to the manufacturer’s instructions.

### *In vitro* cell growth assay

The *in vitro* cell growth assay was carried out as described previously^34^. Briefly, cell viability was measured using the WST-8 assay (Cell Counting Kit-8; Dojindo, Kumamoto, Japan) at the indicated number of days after plating, and the results were normalized to day 1 values. Each assay was carried out in triplicate.

### Treatment with EGFR-TKI

Gefitinib and Erlotinib were purchased from Cayman Chemicals (Michigan, USA) and were resuspended in dimethyl sulfoxide (DMSO) to a final concentration of 100 mM and 500 mM for long-term storage, respectively. Cells were treated with the medium containing Gefitinib (0.001, 0.01, 0.1, 1, 5, 10, 25 µM), Erlotinib (0.001, 0.01, 0.1, 1, 5, 10, 25 µM), or DMSO for 72 hours. Cell viability was assessed by WST-8 assay as described above.

### Gene expression array analysis

Gene expression array analysis was carried out as previously described^36^. Briefly, Panc1, MIAPaCa2, and MDA-MB-231 cells were transfected with 10 nmol/L of miRNA (*miR-NC*, or *miR-3140*). RNA was extracted 72 hours after transfection. The data were analyzed by GeneSpring software (Agilent Technologies, Japan).

### Western blotting

Western blotting was performed according to previously reported methods^36^. Primary antibodies for Western blotting were used as follows: antibodies for BRD4 (#13440), BRD2 (#5848), MYC (#9402), NUT (#3625), Cyclin D2 (#3741 S), p-STAT3 (#9145 S), STAT3 (#9139 S), and CDK6 (#3136) were purchased from Cell Signaling Technology; antibodies for CDK2 (sc-163) and EGFR (sc-03-G) from Santa Cruz Biotechnology; antibodies for BRD3 (A302-368A) from Bethyl Laboratories (Montgomery, TX); ${\rm{\beta }}$-actin (A5441) and Vinculin (V9131) from Sigma-Aldrich (Tokyo, Japan); and HaloTag (G921A) from Promega (Tokyo, Japan).

### Luciferase activity assay

Luciferase reporter plasmids were constructed by inserting the 3′UTR of *CDK2*, *EGFR* and CDS of *BRD4* and *BRD3* downstream of the luciferase gene within the pmirGLO Dual-Luciferase miRNA Target Expression Vector (Promega, Madison, WI, USA). All site-specific mutations used the KOD -Plus- Mutagenesis Kit (TOYOBO, Osaka, Japan). Luciferase reporter plasmids or control plasmid (pmirGLO) were transfected into Panc1 cells using Lipofectamine 2000 (Thermo Fisher Scientific), and 10 nmol/L of miRNA (*miR-NC* or *miR-3140*) was also transfected 6 hours later. After 2 days, Firefly and Renilla luciferase activities were measured using the Dual-Luciferase Reporter Assay System (Promega), and relative luciferase activity was calculated by normalizing the Firefly luciferase reading with its corresponding internal Renilla luciferase control.

### Plasmid construction and transfection

BRD4 and BRD3 cDNA were purchased from Kazusa DNA Research Institute (Chiba, Japan) and were subcloned into the pFN28A HaloTag CMV-neo Flexi Vector (Promega, Madison, WI) to generate a mammalian expression vector. All site-specific mutations were generated using the KOD-Plus-Mutagenesis Kit (TOYOBO). The BRD3 or BRD4 expression vector or the empty vector were transfected into Panc1 and MIAPaCa2 cells using the Lipofectamine 2000 (Thermo Fisher Scientific), according to the manufacturer’s instructions.

### Establishment of JQ1-resistant cells

JQ1-resistant cells derived from Ty-82 were established by long-term incubation with gradually increasing JQ1 concentrations. JQ1 was purchased from APExBIO (Houston, TX). JQ1 was resuspended in DMSO to a final concentration of 10 mM for long-term storage. The cells were initially exposed to JQ1 at 0.1 µmol/L in RPMI medium, then cultured in JQ1-free medium to confluence, and then exposed to JQ1 at a higher concentration. This cycle was repeated several times, until cells that were able to survive in RPMI medium including 2.5 μmol/L JQ1 were defined as Ty-82 JQ1-R cells.

### Real-time xCELLigence impedance analysis of cytotoxicity

Ty-82 cells and Ty-82 JQ1-R cells (3 × 10^4^) were seeded in wells of the E-Plate 16 (ACEA Biosciences, San Diego, CA, USA). Approximately 48 hours later, these cells were treated with JQ1 (0.01, 0.05, 0.1, 0.5, 1, 5, 10 µmol/L) (APExBIO) and DMSO. Cell-electrode impedance was monitored using the xCELLigence RTCA DP system (ACEA Biosciences) to produce time-dependent cell response dynamic curves. Data were collected every 5 min after treatment with JQ1 for the first four hours, every 15 min for the next 20 hours, and then every 1 hour for an additional 48 hours. Dose response curves of JQ1 for Ty-82 cells and Ty-82 JQ1-R cells at 50 hours following treatment with JQ1 were constructed. Cell indexes were normalized with the last time point before treatment with JQ1.

### *In vivo* tumor growth and miRNA administration

*In vivo* miRNA administration of miRNA was performed as previously described^36^. Six-week-old female BALB/c nude mice were purchased from Oriental Bio Service, Japan. Briefly, a total of 5.0 × 10^6^ cells in 100 μL of PBS were subcutaneously injected into the dorsal side of the mice. After tumor formation at day 7, a mixture of 1 nmol dsRNA (*miR-NC* or *miR-3140*) and 100 μL AteloGene (KOKEN, Tokyo, Japan) was administered around the tumor (*miR-NC* to the left dorsal side and *miR-3140* to the right dorsal side of mice). miRNAs were administered on days 7, 11, 15, 18, and 21, and at 23 days after cell injection, mice were sacrificed and tumors were resected. Tumor volume was calculated using the following formula: (shortest diameter)^2^ × (longest diameter) × 0.5. All experimental protocols conducted on the mice were approved by the Tokyo Medical and Dental University Animal Care and Use Committee.

### Immunohistochemistry

Immunohistochemistry was performed as previously described^54^. The resected tumors from xenograft mouse model were fixed in 10% formaldehyde in PBS for 24 h and stored in 70% ethanol and then embedded in paraffin. The following primary antibodies were used for immunohistochemistry: an antibody for BRD4 (HPA061646, 1:500) was purchased from Atlas Antibodies (Stockholm, Sweden), BRD3 (A302-368A, 1:500) and CDK2 (IHC-00374, 1:500) antibodies were from Bethyl Laboratories, and EGFR (sc-03-G, 1:200) antibody was from Santa Cruz Biotechnology (Santa Cruz, CA). BRD4, BRD3, EGFR, and CDK2 staining were scored semiquantitatively using histo-score (H-score) based on staining intensity and percentage of positive cells. Staining intensity was scored as follows: 0 = none, 1 = weak, 2 = moderate, or 3 = strong. H-score was calculated by multiplying the intensity of staining with percentage of cells stained in randomly chosen 3 fields from each specimen^55^.

### Quantitative RT-PCR (qRT-PCR)

Total RNA was extracted using TRIsure reagent (BIOLINE, London, UK) according to the manufacturer’s instructions. For miRNA, total RNA was reverse transcribed using the Taqman Reverse Transcription Kit followed by qRT-PCR performed using Custom Taqman miRNA Assays (Applied Biosystems, Foster City, CA). The miRNA expression was normalized to the internal control *RNU6B*. The following primers were used for the Taqman assay (Thermo Fisher Scientific): human *miR-3140-3p* (244524), *RNU6B* (001093).

### Public datasets

To explore the generality of the miRNA expression and clinical features among pancreatic cancer, breast cancer and acute myeloid leukemia (AML), we examined the public datasets from TCGA (http://cancergenome.nih.gov) retrieved on 24th July 2017. We used TCGA datasets on primary cases for pancreatic cancer, breast cancer and AML, and examined correlation of prognosis and expression of *miR-3140*. The datasets we used included mRNA-expression data on 174 samples of pancreatic cancer, 972 samples of breast cancer, 89 samples of AML and 1881 miRNAs, respectively.

### Statistical analysis

Student’s t-test, Paired *t*-test and log-rank test were performed using R software. P values of < 0.05 were considered significant.

### Ethical Approval

All experimental protocols conducted on the mice were approved by the Tokyo Medical and Dental University Animal Care and Use Committee (0170036C).

## Electronic supplementary material

Supplementary information
