# Publisher Correction: *mi*R-3140 suppresses tumor cell growth by targeting *BRD4* via its coding sequence and downregulates the BRD4-NUT fusion oncoprotein

**DOI:** 10.1038/s41598-018-24861-7

**Published:** 2018-04-25

**Authors:** Erina Tonouchi, Yasuyuki Gen, Tomoki Muramatsu, Hidekazu Hiramoto, Kousuke Tanimoto, Jun Inoue, Johji Inazawa

**Affiliations:** 1 Department of Molecular Cytogenetics, Medical Research Institute, Tokyo Medical and Dental University, Tokyo, Japan; 2 Department of Maxillofacial Surgery, Graduate School, Tokyo Medical and Dental University, Tokyo, Japan; 3 Genome Laboratory, Medical Research Institute, TMDU, Tokyo, Japan; 4 Bioresource Research Center, Tokyo Medical and Dental University, Bunkyo-ku, Tokyo, Japan

Correction to: *Scientific Reports* 10.1038/s41598-018-22767-y, published online 14 March 2018

This Article contains an error in Table 1.

The Mature Sequence data for hsa-miR-3173 is missing. The sequence should read ‘AAAGGAGGAAAUAGGCAGGCCA’.

