# Supplementary material for: miR-3140 suppresses tumor cell growth by targeting BRD4 via its coding sequence and downregulates the BRD4-NUT fusion oncoprotein
Source: Sci Rep. 2018 Mar 14;8:4482. doi: 10.1038/s41598-018-22767-y (PMC5852021; doi:10.1038/s41598-018-22767-y)
Supplement: Supplementary file 1 — Supplementary information [file 41598_2018_22767_MOESM1_ESM.pdf]

miR-3140 suppresses tumor cell growth by targeting _BRD4_ via its coding sequence and downregulates the BRD4-NUT fusion oncoprotein

Erina Tonouchi[1,2], Yasuyuki Gen[1], Tomoki Muramatsu[1], Hidekazu Hiramoto[1], Kousuke Tanimoto[3], Jun Inoue[1], Johji Inazawa[1,4]

Author's affiliations:

[1]Department of Molecular Cytogenetics, Medical Research Institute, Tokyo Medical and Dental University, Tokyo, Japan.

[2]Department of Maxillofacial Surgery, Graduate School, Tokyo Medical and Dental University, Tokyo, Japan.

[3]Genome Laboratory, Medical Research Institute, TMDU, Tokyo, Japan.

[4]Bioresource Research Center, Tokyo Medical and Dental University, Bunkyo-ku, Tokyo, Japan

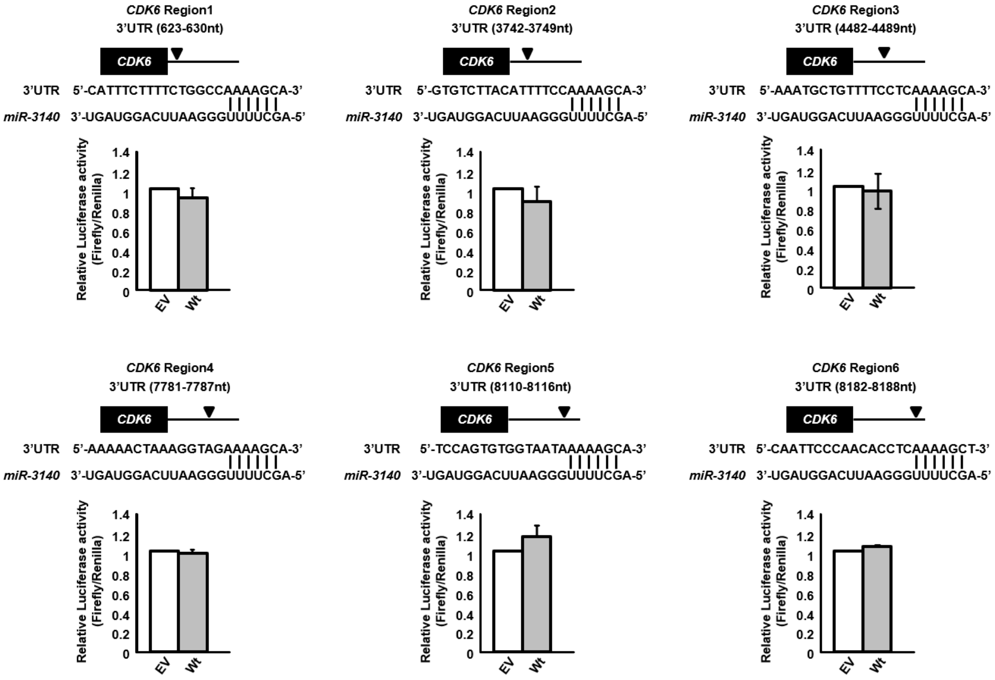

Supplementary Figure S1

Luciferase reporter assays. Panc1 cells were transfected with the pmirGLO Dual Luciferase vectors containing 3’UTR target sites of *CDK6*, and after 6 hours, either *miR-NC* or *miR-3140* was additionally transfected. Top, putative binding site of *miR-3140* within the 3’UTR of *CDK6*. Bottom, results of the luciferase assay.

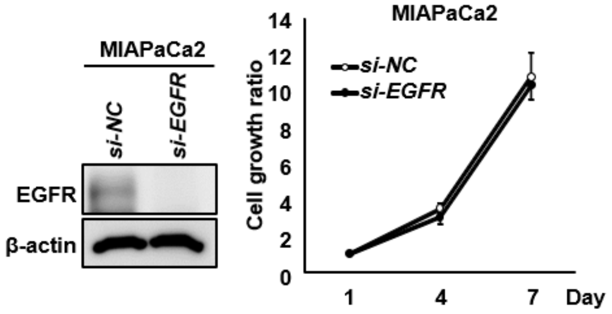

Supplementary Figure S2

Evaluation of the effect of *si-EGFR* in MIAPaCa2 cells. Western blot analysis (left) and cell growth assay (right) in MIAPaCa2 cells after transfection with 20 nmol/L of negative control siRNA (*si-NC*) or siRNA targeting *EGFR*.

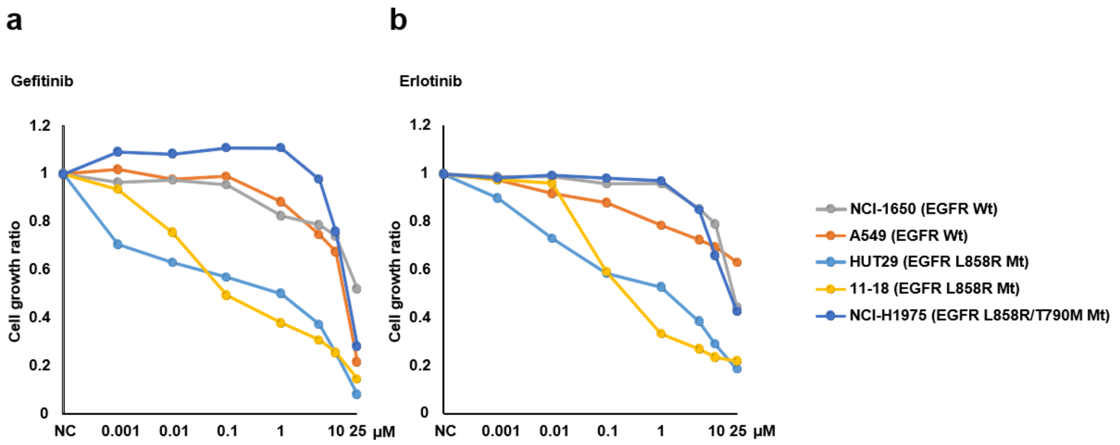

Supplementary Figure S3

Effect of EGFR-TKIs in non-small cell lung carcinoma (NSCLC) cell line.

Dose response curve of Gefitinib (a) and Erlotinib (b) at 72 hours after treatment of each drug in NSCLC cell lines. Cell growth ratio was assessed with WST-8 assay using a relative ratio compared with DMSO-treated cells.

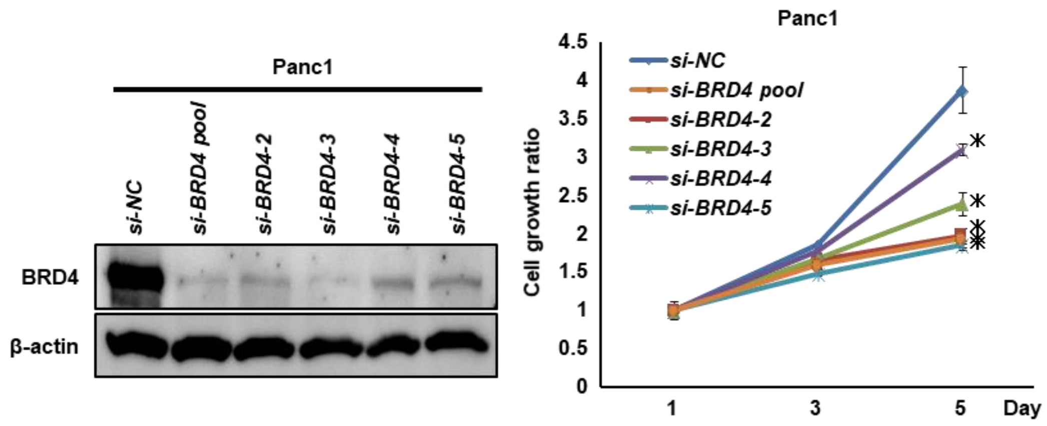

Supplementary Figure S4

Effects of knockdown of *BRD4*. Cells were transfected with 20nmol/L of *si-NC* or siRNAs targeting *BRD4*. Each of *si-BRD4-2*, *3*, *4*, or *5* is a single siRNA duplex and *si-BRD4*-pool consists of all 4 siRNAs. Left, western blot analysis of indicated proteins in Panc1 cells 72 hours after transfection. Right, results of the cell growth assay. The cell growth ratio was assessed with the WST-8 assay based on the relative ratio compared with day 1. Bar, SD for triplicate experiments; *$P < 0.05$.

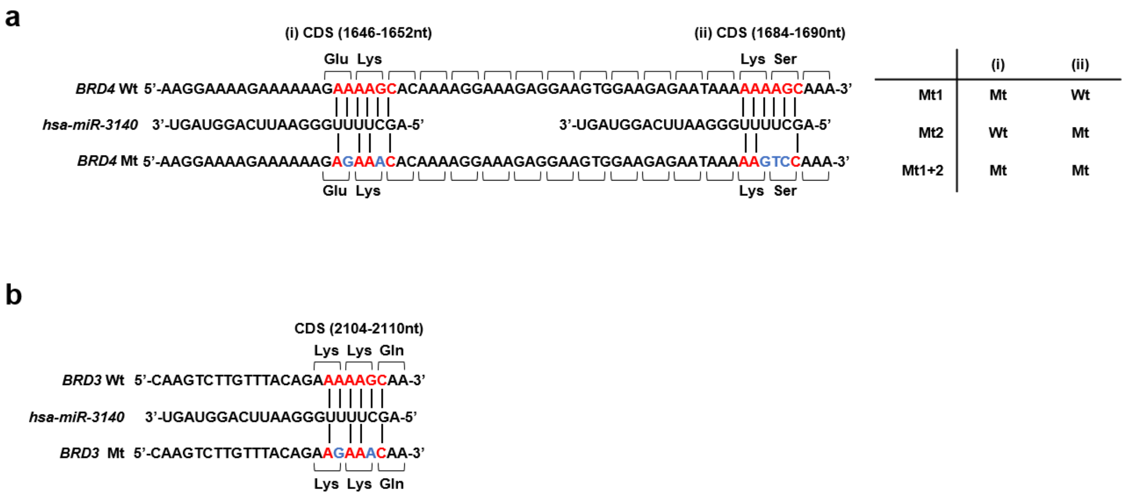

Supplementary Figure S5

Synonymous mutation sequence of BRD4 and BRD3 expression vector.

Putative binding site of *miR-3140* within CDS of *BRD4* (a) and *BRD3* (b), and synonymous mutation sequences.

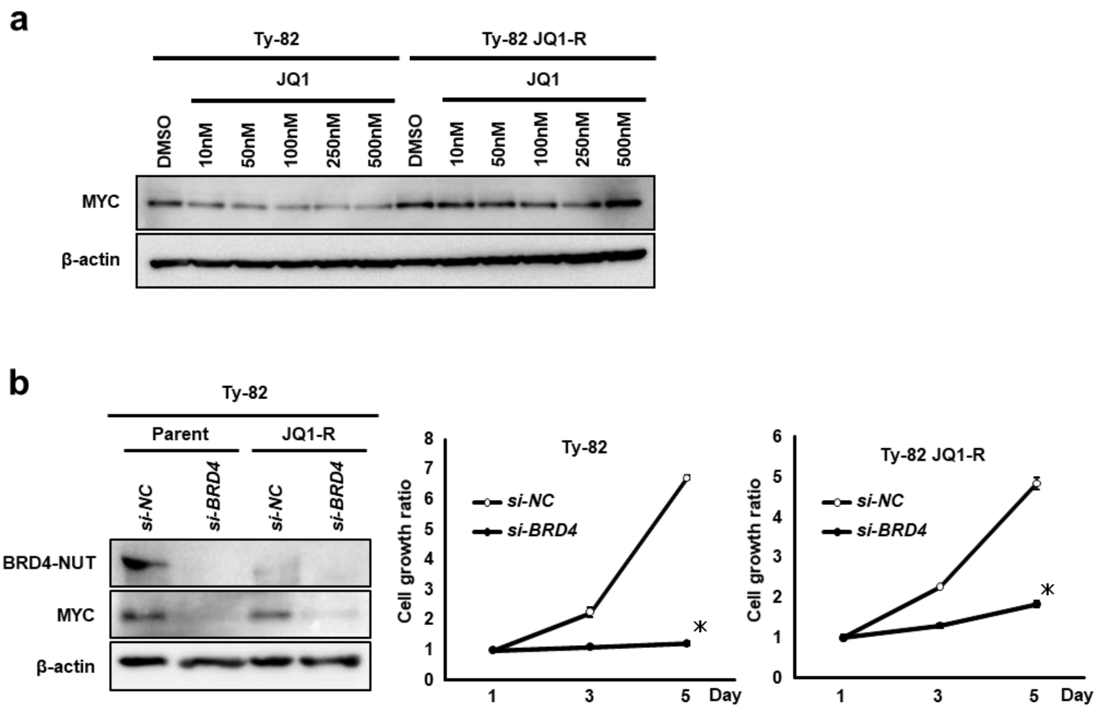

Supplementary Figure S6

(a) Effects of the BET inhibitor JQ1 in Ty-82 and its JQ1-resistant (Ty-82 JQ1-R) cells. Western blot analysis of MYC in Ty-82 and Ty-82 JQ1-R cells 3 hours after JQ1 treatment.

(b) Effects of knockdown of BRD4-NUT in Ty-82 and Ty-82 JQ1-R cells. Cells were transfected with 20 nmol/L of *si-NC* or siRNA targeting *BRD4*. The binding sequences of pooled siRNAs targeting *BRD4* exist within *BRD4-NUT* fusion gene. Left, western blot analysis of indicated proteins in Ty-82 and Ty-82 JQ1-R cells 72 hours after transfection. Right, results of the cell growth assay. The cell growth ratio was assessed with the WST-8 assay based on the relative ratio compared with day 1. Bar, SD for triplicate experiments; *$P < 0.05$.

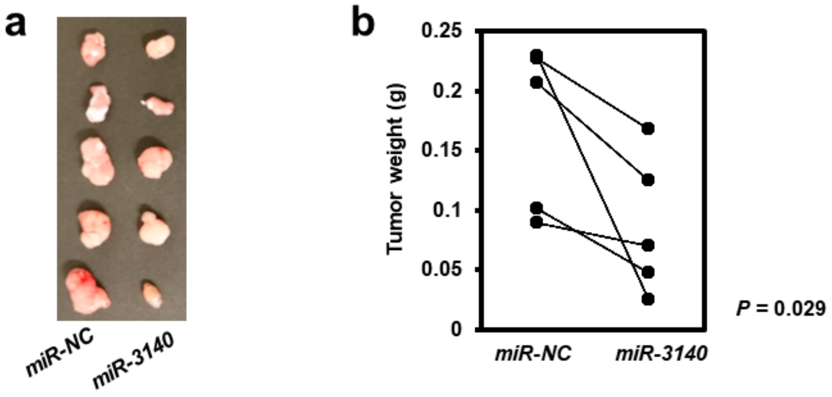

Supplementary Figure S7

(a) The representative image of resected tumors from *miR-NC* or *miR-3140* treated mice.

(b) Differences in tumor weights between tumors treated with *miR-NC* and those treated with *miR-3140* in each mouse. One-tailed paired *t*-test was used for comparison of weights of the resected tumors in each mouse (P = 0.029).

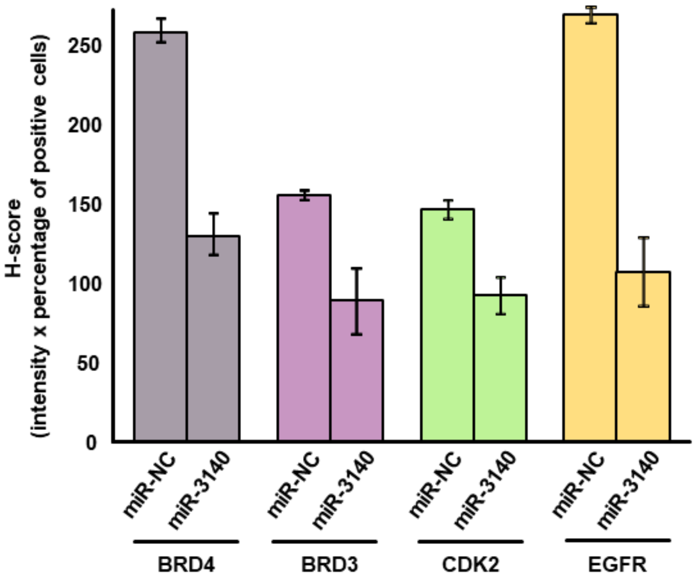

Supplementary Figure S8

Evaluation of immunohistochemistry for the targets of *miR-3140*. Histo-score (H-score) was calculated by multiplying the intensity of staining with percentage of cells stained (see Materials and Methods section).

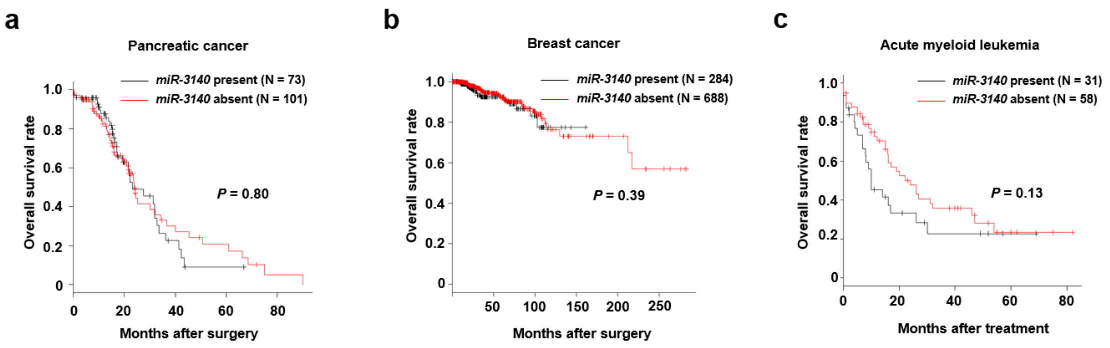

Supplementary Figure S9

Kaplan-Meier curves for overall survival rates of patients with primary acute myeloid leukemia, breast cancer and pancreatic cancer in TCGA data. The expression of *miR-3140* is not correlated with overall survival in a corresponding cohort of 174 patients with pancreatic cancer (a), 972 patients with breast cancer (b), and 89 patients with acute myeloid leukemia (c) in TCGA database. ($P = 0.80$, $P = 0.38$, and $P = 0.13$, log-rank test, respectively).

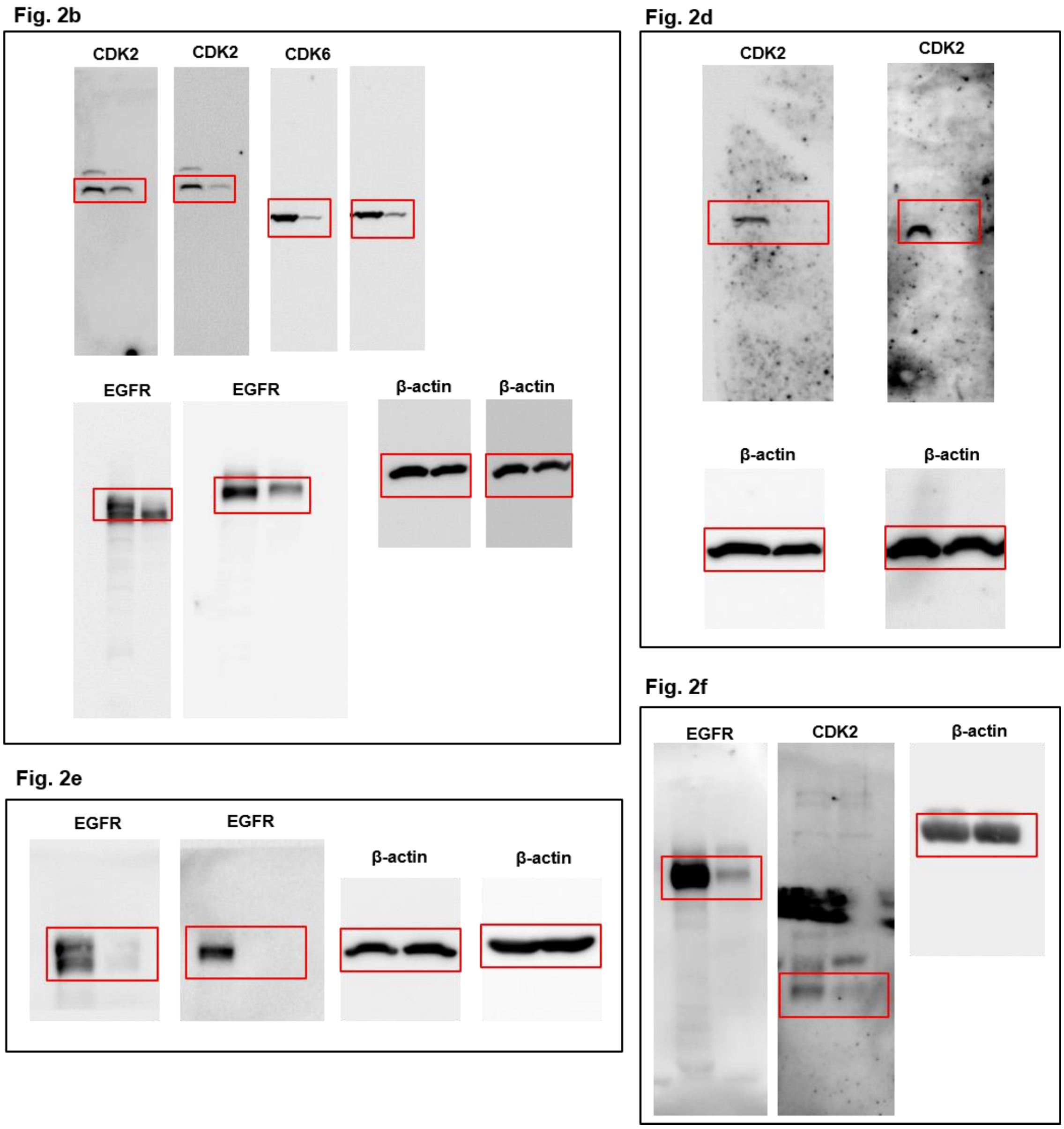

Supplementary Figure S10. Full-length blots for Figure 2b, 2d, 2e and 2f.

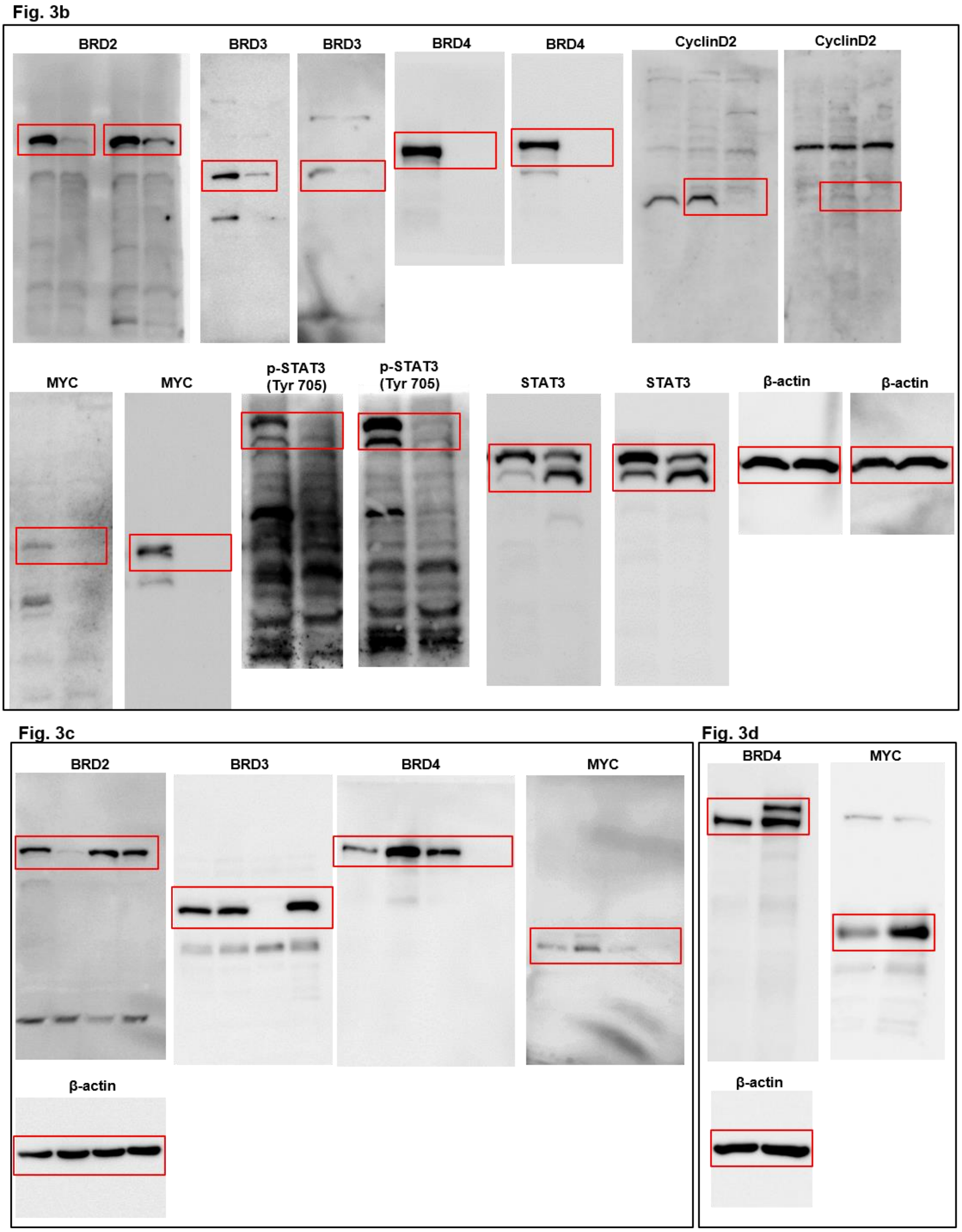

Supplementary Figure S11. Full-length blots for Figure 3b, 3c and 3d.

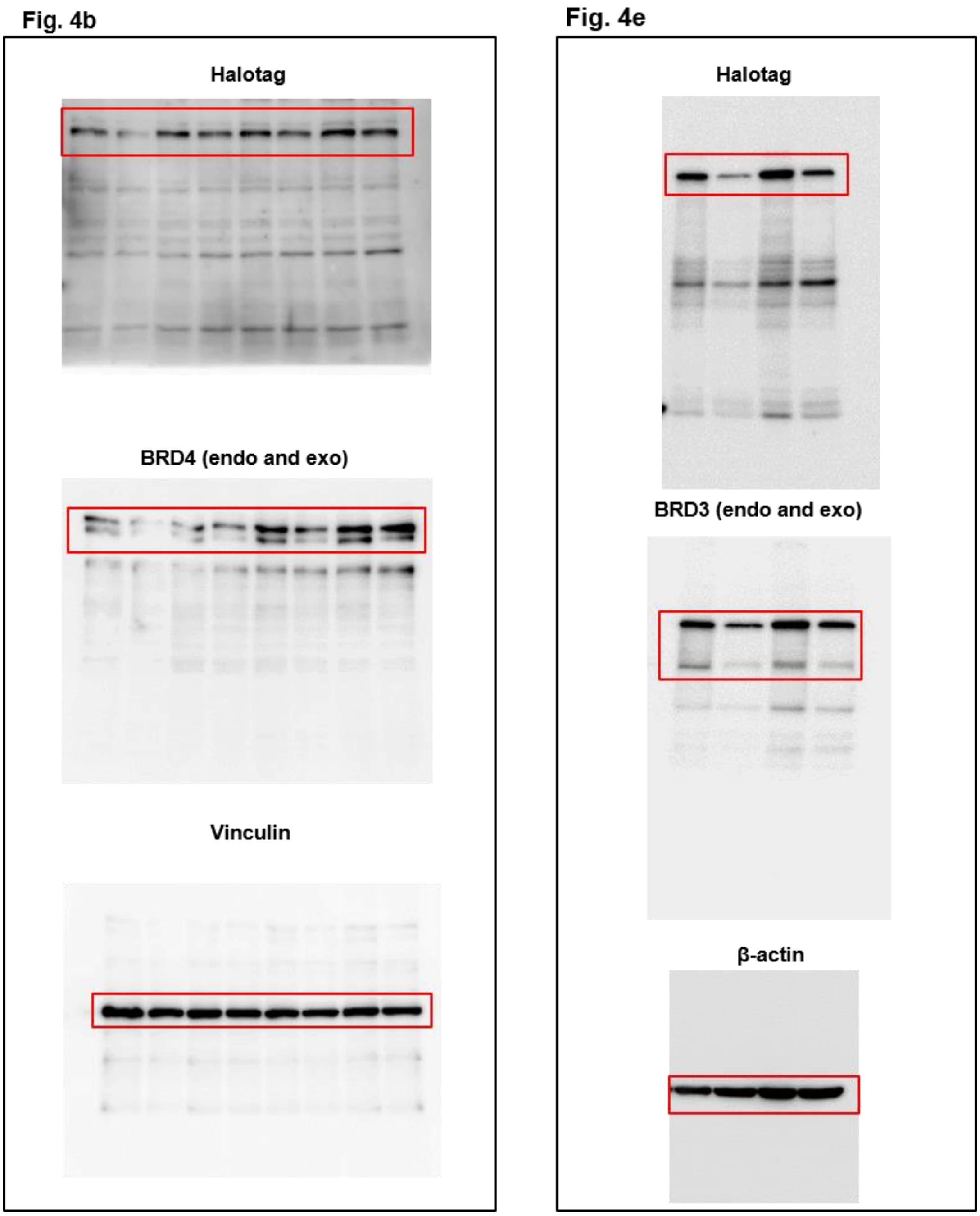

Supplementary Figure S12. Full-length blots for Figure 4b and 4e.

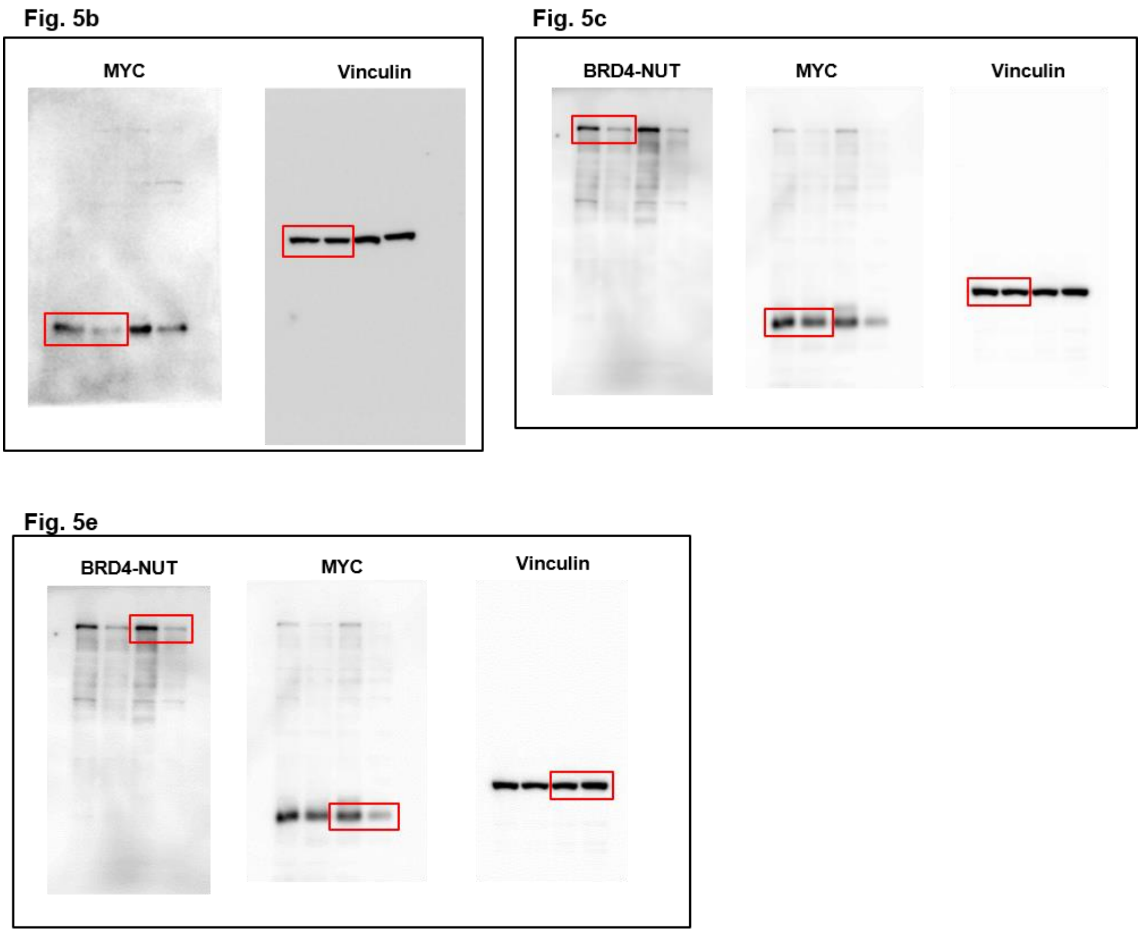

Supplementary Figure S13. Full-length blots for Figure 5b, 5c, and 5e.

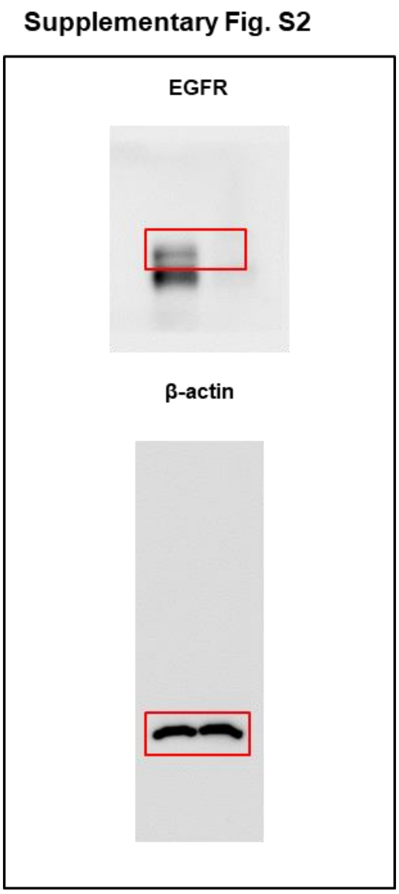

Supplementary Figure S14. Full-length blots for Supplementary Figure S2.

Supplementary Fig. S4

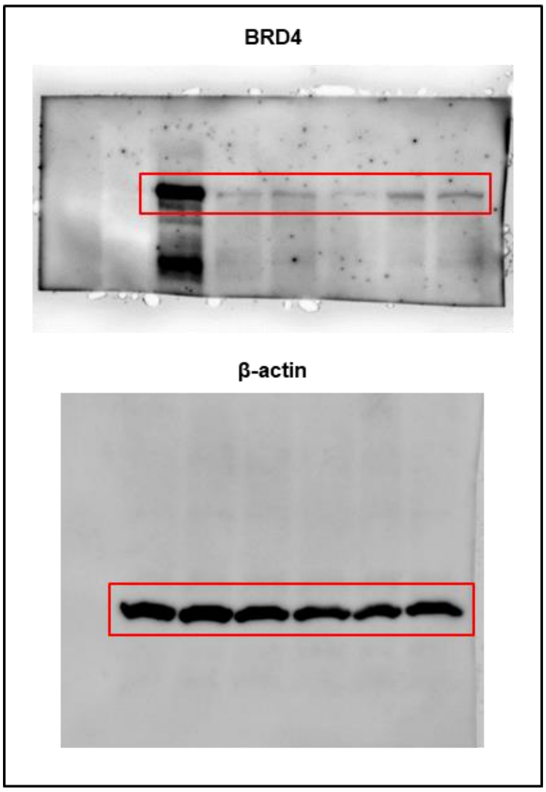

Supplementary Figure S15. Full-length blots for Supplementary Figure S4.

Supplementary Fig. S6

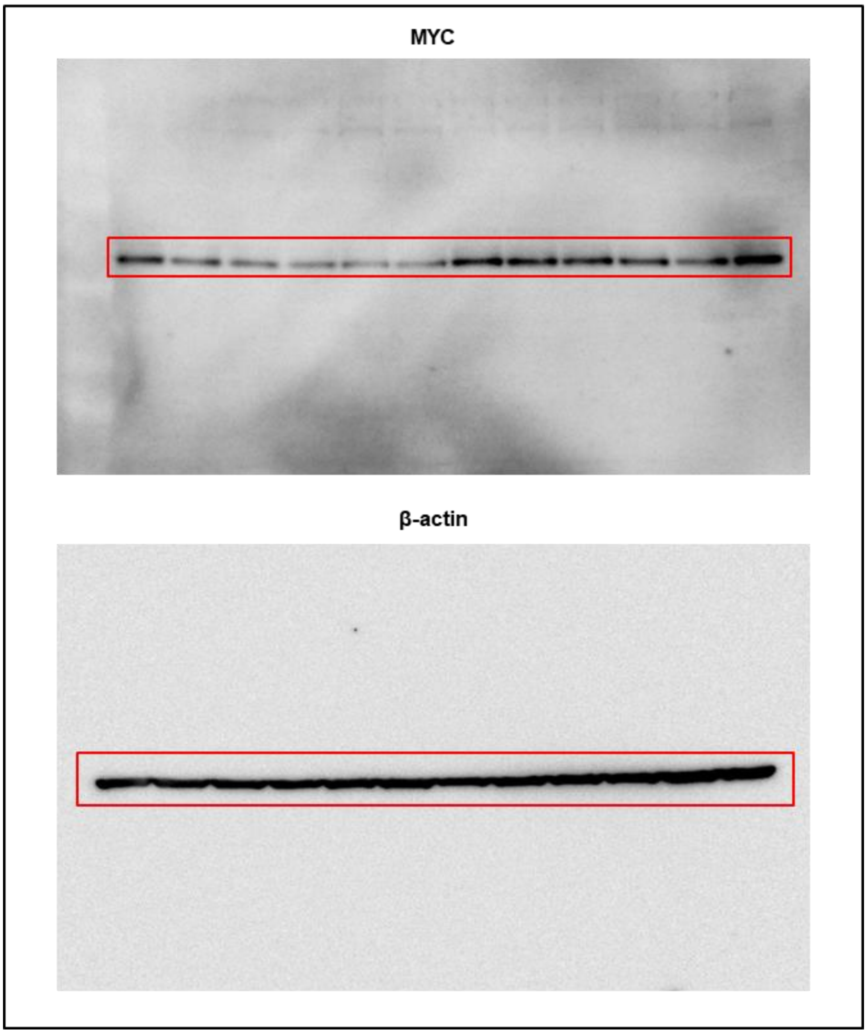

Supplementary Fig. S6b

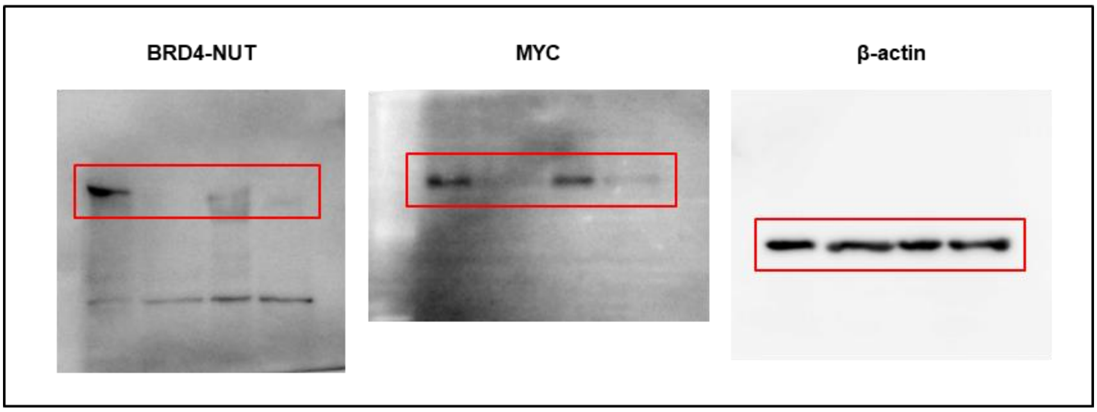

Supplementary Figure S16. Full-length blots for Supplementary Figure S6.

Supplementary Table S1. The gene list of 103 genes which are potential CDS targets of *miR-3140*.

ANAPC1	*ANLN*	*ASNS*	*ASPH*	*BAIAP2L1*	*BIRC5*	*BRD4*	*C10orf12*
CCNB2	*CDCA2*	*CENPN*	*CHEK2*	*CHORDC1*	*CLEC2D*	*CLIC4*	*COG2*
CREBRF	*CSNK2A2*	*CWF19L1*	*DIAPH3*	*DNAJC14*	*DNAJC3*	*EIF4EBP2*	*EPB41*
EPB41L4B	*FAM204A*	*FAM217B*	*FAM220A*	*FAM222B*	*FNBP1*	*FOXN3*	*GAS2L3*
GOLGA6L9	*GTSE1*	*HIRIP3*	*HJURP*	*HN1L*	*HSD17B7*	*IDH2*	*IMMT*
IWS1	*KIF20A*	*KIF23*	*KIF2C*	*KLF11*	*LARP1B*	*LCOR*	*LCORL*
LIN9	*LRRC8B*	*MAML1*	*MAP3K3*	*MAP4*	*MBTD1*	*MIS18A*	*MRPS27*
MSH5	*MTIF2*	*MTL5*	*MYH9*	*NEK2*	*NF2*	*NFXL1*	*NOLC1*
NUDT21	*NUF2*	*OSMR*	*PDCL*	*PHACTR4*	*PKN2*	*PLCE1*	*POLH*
POLQ	*PRR13*	*RAB11FIP1*	*RALBP1*	*RBM41*	*RBMS1*	*RPL14*	*SFSWAP*
SMC4	*SNX27*	*SNX3*	*SORBS1*	*SPAG5*	*SPC25*	*STIL*	*TAB2*
TACC1	*TAF1B*	*TAF4B*	*TERF1*	*TET1*	*TMEM52*	*TTC1*	*TTK*
TTPAL	*ULK2*	*USP32*	*YIPF4*	*ZFP64*	*ZNF254*	*ZNF318*	
